# Leveraging health records to identify diagnoses associated with recurrent pregnancy loss across two medical centers

**DOI:** 10.1016/j.isci.2026.114633

**Published:** 2026-01-07

**Authors:** Jacquelyn Roger, Feng Xie, Jean M. Costello, Alice S. Tang, Jay Liu, Tomiko T. Oskotsky, Sarah R. Woldemariam, Idit Kosti, Brian L. Le, Michael P. Snyder, Linda C. Giudice, Gary M. Shaw, David K. Stevenson, Aleksandar Rajkovic, M. Maria Glymour, Dara Torgerson, Nima Aghaeepour, Hakan Cakmak, Ruth B. Lathi, Marina Sirota

**Affiliations:** 1 Biological and Medical Informatics Graduate Program, University of California, San Francisco, San Francisco, CA, USA; 2 Bakar Computational Health Sciences Institute, University of California, San Francisco, San Francisco, CA, USA; 3 Department of Anesthesiology, Perioperative, and Pain Medicine, Stanford University, Stanford, CA, USA; 4 Department of Pediatrics, Stanford University, Stanford, CA, USA; 5 Department of Biomedical Data Science, Stanford University, Stanford, CA, USA; 6 Department of Genetics, Stanford University School of Medicine, Stanford, CA, USA; 7 Department of Obstetrics and Gynecology, University of California, San Francisco, San Francisco, CA, USA; 8 Department of Pathology, University of California, San Francisco, San Francisco, CA, USA; 9 Institute of Human Genetics, University of California, San Francisco, San Francisco, CA, USA; 10 Department of Epidemiology and Biostatistics, University of California, San Francisco, San Francisco, CA, USA; 11 Department of Obstetrics and Gynecology, Stanford University, Stanford, CA, USA

**Keywords:** Health sciences, Medicine

## Abstract

Recurrent pregnancy loss (RPL), defined as 2 or more pregnancy losses, affects 5–6% of ever-pregnant individuals. Approximately half of these cases have no identifiable explanation. In this study, we aim to identify diagnoses associated with RPL and generate hypotheses about RPL etiology utilizing electronic health record (EHR) data. We implemented a case-control study comparing the history of over 1,600 diagnoses between RPL and live birth patients, leveraging the University of California San Francisco (UCSF) and Stanford University EHR databases. In total, our study includes 8,496 RPL (UCSF: 3,840, Stanford: 4,656) and 53,278 control (UCSF: 17,259, Stanford: 36,019) patients. Menstrual abnormalities and infertility-associated diagnoses are significantly positively associated with RPL in both medical centers. Age-stratified analysis revealed that the majority of RPL-associated diagnoses have higher odds ratios for patients <35 years compared with 35+ years patients. While Stanford results are sensitive to control for healthcare utilization, UCSF results are stable across analyses with and without utilization.

## Highlights

• Etiology of recurrent pregnancy loss, defined as 2 or more pregnancy losses, remains elusive
• EHR data from two medical centers were analyzed to generate hypotheses about RPL etiology
• Menstrual abnormalities and infertility diagnoses are significantly associated with RPL
• RPL-associated diagnoses have higher odds ratios for patients under 35 years of age

## Graphical abstract

**Figure:**
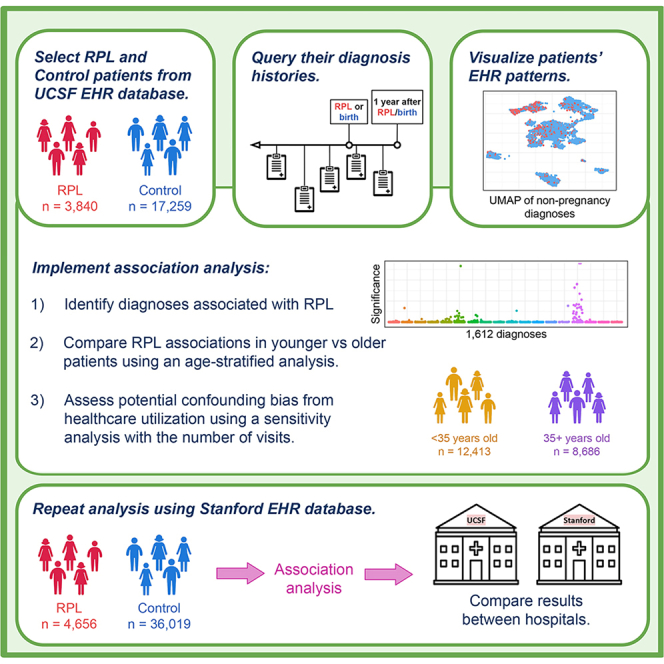

## Introduction

Pregnancy loss is an umbrella term that encompasses any pregnancy that does not progress to live birth. It includes both miscarriage and stillbirth, which are differentiated by whether the demise occurs before or after 20 weeks’ gestation according to US diagnostic criteria.^1^^,^^2^^,^^3^ Pregnancy loss is one of the most common pregnancy complications, occurring in almost a third of all clinically recognizable pregnancies.^4^^,^^5^^,^^6^ Recurrent pregnancy loss (RPL), defined as two or more losses, is also common; an estimated 5–6% of ever-pregnant individuals and up to 16% of parous individuals experience RPL.^7^^,^^8^ RPL can be devastating for pregnant individuals and their families^9^^,^^10^^,^^11^ and is associated with a poorer prognosis for subsequent pregnancies.^12^

RPL is multifactorial. Known risk factors include maternal age over 35 years, maternal or fetal numerical or segmental chromosomal abnormalities, maternal congenital uterine abnormalities, maternal antiphospholipid antibody syndrome, and uncontrolled maternal hormonal or metabolic conditions.^13^^,^^14^^,^^15^^,^^16^^,^^17^^,^^18^^,^^19^ All of these risk factors are commonly screened in RPL clinical workups regardless of the gestational age of the losses. However, for some of these factors, their relative contribution to risk of early vs. later loss differs. For example, cytogenic abnormalities are present in roughly half of miscarriages but <15% of stillbirths.^20^ Several hypothesis-driven studies have uncovered additional associations with RPL, including lifestyle factors,^21^^,^^22^^,^^23^ genetic variation,^24^^,^^25^^,^^26^^,^^27^^,^^28^^,^^29^ infertility-associated diagnoses,^30^^,^^31^^,^^32^ hereditary thrombophilias,^33^^,^^34^^,^^35^ infections,^36^^,^^37^^,^^38^ and environmental exposures.^39^^,^^40^^,^^41^^,^^42^^,^^43^ There is also increasing evidence of the role of paternal health in pregnancy loss,^44^ including sperm DNA fragmentation.^45^^,^^46^ Still, approximately half of all RPL patients have no identifiable causes for their losses.^47^^,^^48^^,^^49^ This substantial gap suggests that our current understanding of RPL etiologies is incomplete.

Electronic health record (EHR) databases contain multimodal longitudinal data on patients. Computational methods provide the opportunity to mine these data and uncover patterns within patient populations in a hypothesis-agnostic manner. Previous projects analyzing EHR data to study RPL have investigated disease incidence after RPL^50^ and characterized lifetime phenotypic associations of idiopathic RPL.^24^ The first study included 10,691 RPL patients from Denmark and reported an increased risk of cardiovascular and gastrointestinal disorders later in life, for primary and secondary RPL patients, respectively.^50^ The second study included 458 idiopathic RPL patients from the United Kingdom Biobank (UKBB) and reported that patients with idiopathic RPL were more likely to be diagnosed with tubulointerstitial nephritis, infertility, or ectopic pregnancy anytime in their lifetimes.^24^

To our knowledge, there have not been any comprehensive EHR studies focused on identifying diagnoses occurring before or near RPL onset across a diverse population of patients. This could uncover potential risk factors for RPL and open up new avenues for RPL management research.

With these objectives in mind, we implemented a case-control study comparing the frequency of candidate diagnoses in RPL and live birth patients, leveraging deidentified EHR data from the University of California San Francisco (UCSF) and Stanford University databases. All diagnoses occurring before RPL onset (or first live birth for control patients) up until a year following RPL onset (or birth) were considered. The year afterward was included because sometimes patients undergo diagnostic evaluations following RPL (or birth) that are relevant for understanding their pregnancy outcomes. To compare RPL associations in younger vs. older patients, we carried out an age-stratified analysis of patients under 35 years of age and patients 35 years and older. In addition, we hypothesized that RPL patients may have increased healthcare utilization relative to control patients, which could lead to increased recognition of pregnancies and incidental diagnoses. We therefore conducted a sensitivity analysis controlling for the number of visits as an indicator of utilization. Lastly, to ascertain which results validated across medical centers, we compared findings from our UCSF and Stanford studies. This validation work builds on our existing foundation of porting EHR analysis models between medical centers toward generalizability of both methods and results.^51^

**Figure 1:**
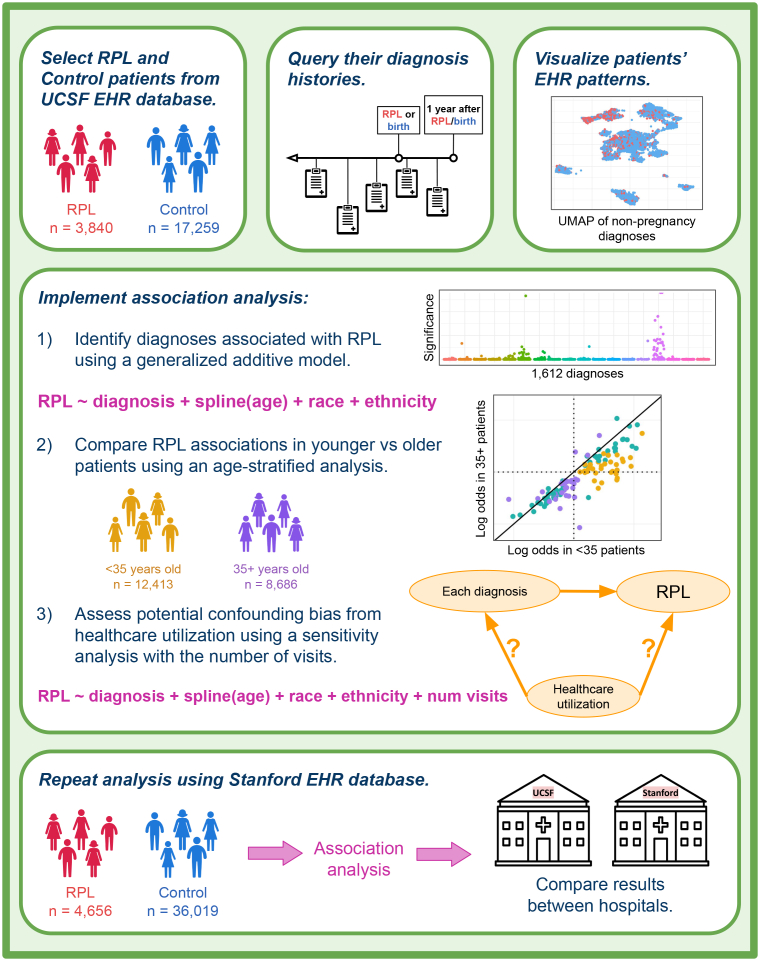
Study design Patients were selected from the UCSF OMOP EHR database by querying for records of RPL (for RPL group) or live birth (for control group). For all patients selected, we queried their diagnosis histories prior to their first record of RPL/birth up until a year afterward. We visualized patients’ EHR patterns using a UMAP of their non-pregnancy-related diagnoses. Three analyses were implemented: (1) main association analysis, (2) age-stratified analysis, and (3) healthcare utilization sensitivity analysis. All queries and analyses were repeated using the Stanford EHR database for external validation.

Here, we aim to identify diagnoses associated with RPL and generate hypotheses about RPL etiology through exploration of EHR data (Figure 1). We compare the diagnostic histories of RPL patients and control patients from two independent medical centers and assess the sensitivity of our association results to age, healthcare utilization, length of EHRs, and RPL severity.

## Results

### Description of RPL and control patients

**Figure 2:**
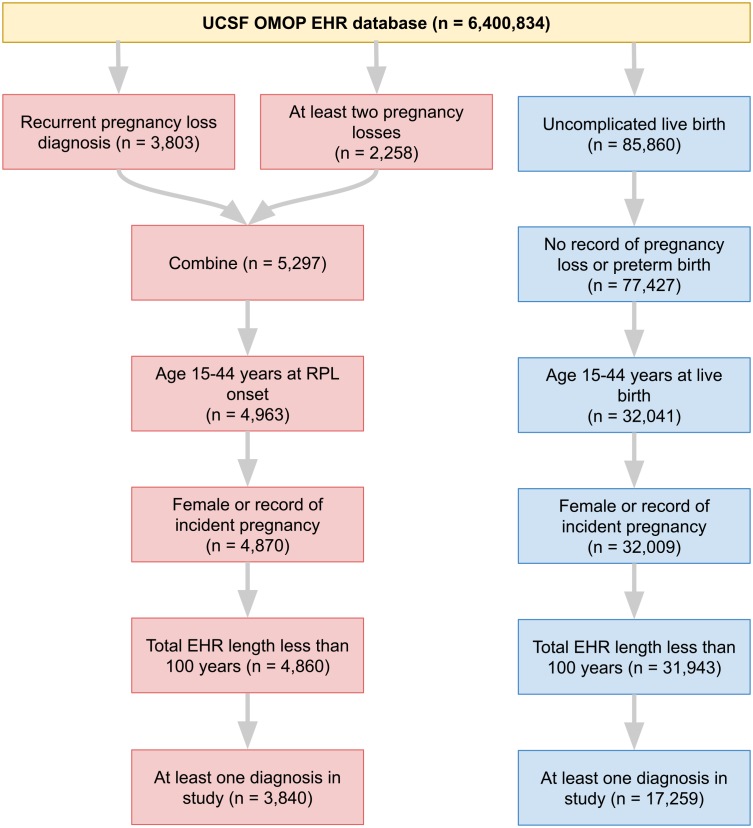
Patient selection at UCSF In total, 3,840 RPL patients were selected (red) and 17,259 control patients were selected (blue).

We identified RPL and control patients in the UCSF and Stanford deidentified EHR databases by querying for concepts indicating pregnancy losses or live birth (see STAR Methods, Data S1). The UCSF and Stanford EHR databases consist of patients who have had at least one interaction (e.g., visit, diagnosis, procedure) with the respective medical centers. From the UCSF EHR database containing 6.4 million patients, 3,840 RPL and 17,259 control patients were selected. From the Stanford EHR database containing 3.6 million patients, 4,656 RPL and 36,019 control patients were selected. The patient inclusion and exclusion criteria are summarized in flowcharts for UCSF patients (Figure 2) and Stanford patients (Figure S1).

UCSF patients’ demographics and healthcare utilization distributions are summarized in Table 1. Overall, RPL patients are older than control patients (median age: 36.6 vs. 33.4, t test *p*-value < 0.001). Fewer RPL patients are identified as Hispanic or Latino compared with control patients (11.7% vs. 16.6%, chi-squared test *p*-value < 0.001). RPL patients have slightly more visits (median 42.5 vs. 41, t test *p*-value < 0.001), longer EHR records (median 3.44 vs. 2.04 years, t test *p*-value < 0.001), and fewer unique diagnoses (median 9 vs. 13, t test *p*-value < 0.001).

**Table 1:** UCSF patients

	RPL	Control	Total	*p*-value
	(*N* = 3840)	(*N* = 17259)	(*N* = 21099)	
**Age**				

Mean (SD)	36.2 (4.53)	33.0 (5.09)	33.6 (5.15)	<0.001
Median [Min, Max]	36.6 [16.6, 44.0]	33.4 [15.1, 44.0]	34.0 [15.1, 44.0]	–

**Race**				

American Indian or Alaska Native	33 (0.9%)	86 (0.5%)	119 (0.6%)	<0.001
Asian	825 (21.5%)	3872 (22.4%)	4697 (22.3%)	–
Black or African American	180 (4.7%)	878 (5.1%)	1058 (5.0%)	–
Native Hawaiian or Other Pacific Islander	26 (0.7%)	145 (0.8%)	171 (0.8%)	–
Other Race	570 (14.8%)	3401 (19.7%)	3971 (18.8%)	–
Unknown	366 (9.5%)	1043 (6.0%)	1409 (6.7%)	–
White	1840 (47.9%)	7834 (45.4%)	9674 (45.9%)	–

**Ethnicity**				

Hispanic or Latino	450 (11.7%)	2869 (16.6%)	3319 (15.7%)	<0.001
Not Hispanic or Latino	3022 (78.7%)	13266 (76.9%)	16288 (77.2%)	–
Unknown	368 (9.6%)	1124 (6.5%)	1492 (7.1%)	–

**Number of visits (in study)**				

Mean (SD)	67.3 (79.8)	53.1 (52.3)	55.7 (58.5)	<0.001
Median [Min, Max]	42.5 [1.00, 1700]	41.0 [1.00, 1230]	41.0 [1.00, 1700]	–

**Years in EHR (in study)**				

Mean (SD)	5.99 (6.46)	4.87 (5.89)	5.08 (6.01)	<0.001
Median [Min, Max]	3.44 [0.137, 44.4]	2.04 [0.132, 41.4]	2.23 [0.132, 44.4]	–

**Number of diagnoses (in study)**				

Mean (SD)	13.7 (15.2)	15.1 (11.7)	14.8 (12.4)	<0.001
Median [Min, Max]	9.00 [1.00, 202]	13.0 [1.00, 186]	12.0 [1.00, 202]	–

Stanford patients’ demographics and healthcare utilization distributions are summarized in Table S1. Overall, RPL patients are older than control patients (median age 35.4 vs. 32.4, t test *p*-value < 0.001). Fewer RPL patients are identified as Hispanic or Latino compared with control patients (16.4% vs. 29.3%, chi-squared test *p*-value < 0.001). RPL patients have more visits (median 31 vs. 14, t test *p*-value < 0.001), longer EHR records (median 3.14 vs. 1.67 years, t test *p*-value < 0.001), and more unique diagnoses (median 11 vs. 9, t test *p*-value < 0.001).

**Figure 3:**
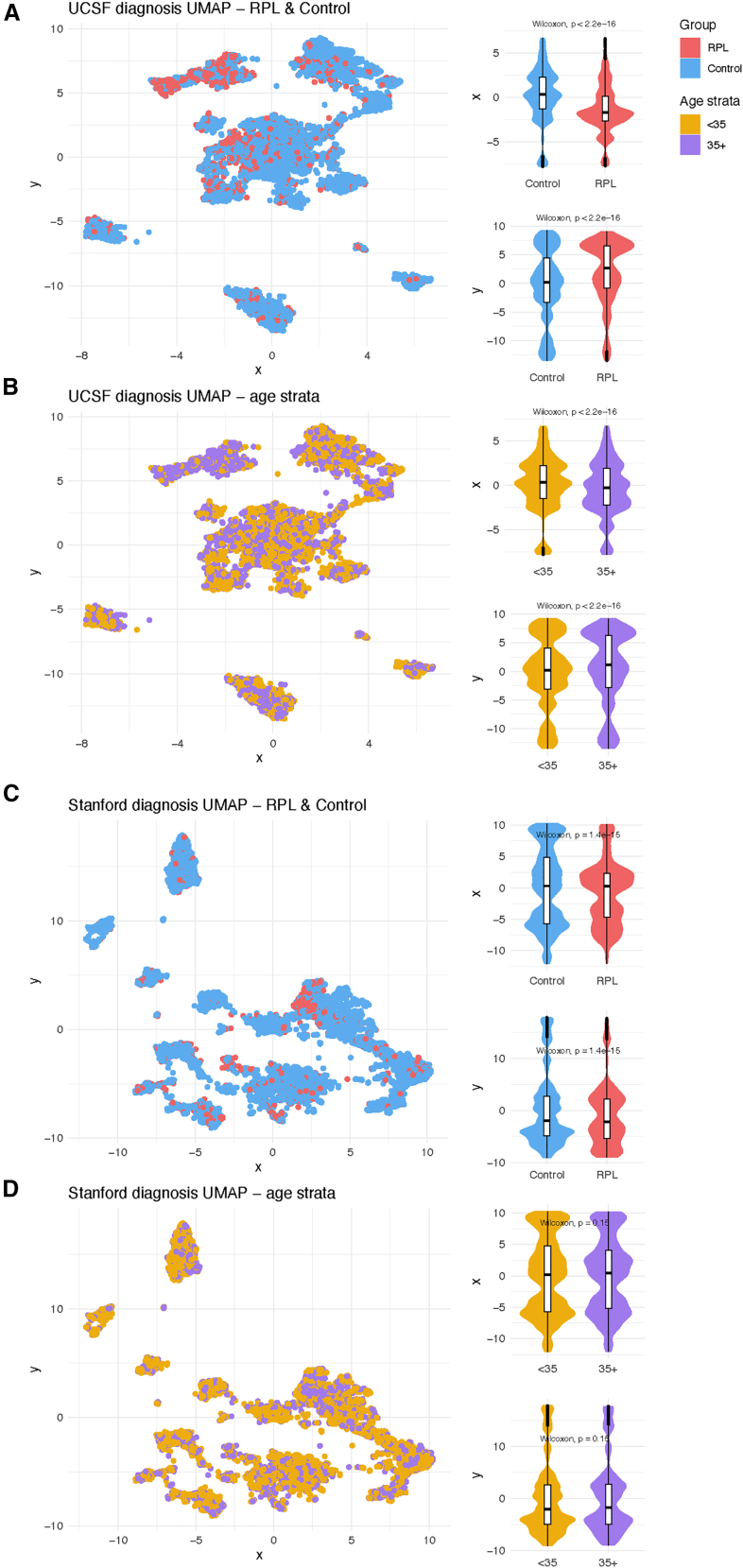
UMAP of non-pregnancy diagnoses We applied UMAP (a dimensionality reduction algorithm) to visualize each patient’s diagnosis patterns and compared dimensions using violin/boxplots at (A) UCSF, (B) UCSF stratified by age, (C) Stanford, and (D) Stanford stratified by age. Diagnoses in the “pregnancy complications” category were excluded from the UMAP to ensure that the subsequent dimensionality reduction was based on diagnoses leading to the outcome (RPL or live birth), instead of diagnoses indicating the outcome.

To visualize RPL and control patients’ EHR patterns, we applied Uniform Manifold Approximation and Projection (UMAP) to their non-pregnancy diagnoses and then plotted the patients’ coordinates in two-dimensional space. UMAP coordinate distributions are significantly different between UCSF RPL and control patients (Wilcoxon rank-sum test, *p* < 0.001, Figure 3A) and between UCSF age strata (Wilcoxon rank-sum test, *p* < 0.001, Figure 3B). For Stanford patients, UMAP coordinate distributions also differ significantly between RPL and control patients (Wilcoxon rank-sum test, *p* < 0.001, Figure 3C) but not between age strata (Wilcoxon rank-sum test, *p* = 0.15, Figure 3D). UCSF and Stanford UMAP plots visualized across patients’ race, ethnicity, number of visits, years in EHR, and number of diagnoses are included in Figures S2–S5.

### Association analysis identifies 88 diagnoses that are statistically significantly associated with RPL at both UCSF and Stanford

For the association analysis, we evaluated whether each candidate diagnosis was positively or negatively associated with RPL. Positive associations suggest potential risk factors for RPL. Negative associations either suggest potential protective factors for RPL or reflect diagnoses that occur more frequently in live birth (control) patients.

Any diagnosis occurring at least once in an RPL or control patient’s EHR during the study window was included as a candidate diagnosis. ICD diagnostic codes were mapped to Phecodes (see STAR Methods) resulting in 1,612 and 1,662 candidate diagnoses at UCSF and Stanford, respectively. Each candidate diagnosis was tested for its association with RPL using a generalized additive model (GAM), with maternal age, race, and ethnicity included as covariates. In each association analysis, all *p*-values were adjusted for multiple testing using the Benjamini-Hochberg method to control the false discovery rate.

**Figure 4:**
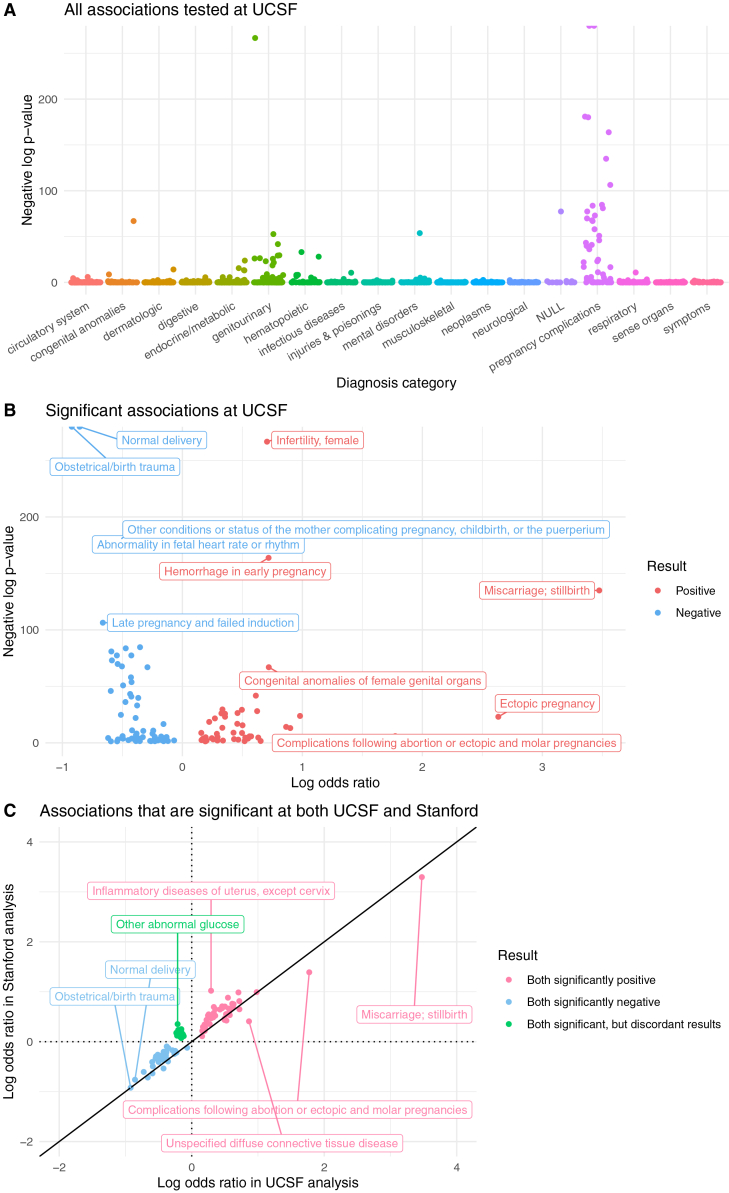
UCSF association analysis results (A) Manhattan plot of all diagnoses tested in the UCSF analysis. Diagnosis categories are listed on the x axis. The y axis is the negative log of each associations’ *p*-value, from each diagnosis’s GAM. The NULL-labeled category contains diagnoses that were not part of any of the other categories listed. (B) Volcano plot of significant (*p*-value < 0.05) associations. In the Manhattan and volcano plots, a few diagnoses have negative log *p*-values that are approaching infinity, so their corresponding points are located on the top border of the plot. (C) Log-log plot of validated positive, validated negative, and discordant association results. These diagnoses are significant in both the UCSF and Stanford analyses. All points in the log-log plots were filtered to include diagnoses where ≥10 patients in either the RPL or control group have a record of that diagnosis. All log transformations in these plots are in base 10.

At UCSF, 1,612 candidate diagnoses were tested (Figure 4A, Data S2); 120 have a statistically significant relationship (adjusted *p*-value < 0.05) with RPL: 51 diagnoses are positively associated with RPL and 69 are negatively associated with RPL (Figure 4B). At Stanford, 1,662 diagnoses were tested (Figure S6A, Data S3); 367 show a statistically significant relationship with RPL: 330 positive and 37 negative (Figure S6B). Confidence intervals from the UCSF and Stanford analyses are reported in Data S4 and S5. Overall, Stanford has a higher proportion of positive associations than UCSF (330/367 vs. 51/120, respectively).

In total, 1,576 candidate diagnoses were shared across the UCSF and Stanford analyses. To determine which results validated across medical centers, we computed the intersection of statistically significant results from the UCSF and Stanford analyses. We found that 88 diagnoses are statistically significantly associated with RPL at both sites, and their odds ratios are very highly correlated between sites (Spearman r = 0.946, *p*-value < 0.001). Of those 88 diagnoses, 42 diagnoses are positive in both, 34 are negative in both, and 12 are discordant (significant in both, but in opposite directions).

### Menstrual abnormalities and infertility-associated diagnoses are positively associated with RPL

Inter-center validated positive association results are summarized in Table 2. At both UCSF and Stanford, we replicated known RPL associations including chromosomal anomalies, congenital anomalies of genital organs, primary hypercoagulable state, and hemorrhage in early pregnancy. Our association analysis also uncovered several menstrual abnormalities: absent/infrequent menstruation (UCSF odds ratio: 1.67, Stanford odds ratio: 2.74), excessive/frequent menstruation (UCSF odds ratio: 1.94, Stanford odds ratio: 2.97), irregular menstrual cycle/bleeding (UCSF odds ratio: 2.67, Stanford odds ratio: 4.41), and other menstrual disorders. All inter-center validated menstrual abnormality associations have *p* < 0.001 from their respective GAMs.

**Table 2:** Positive associations

Diagnosis	UCSF odds ratio	UCSF *p*-value	Stanford odds ratio	Stanford *p*-value
**(a) Known RPL associations**				

Cervical incompetence	2.87	<0.001	4.68	<0.001
Chromosomal anomalies	3.14	<0.001	2.91	<0.001
Chronic lymphocytic thyroiditis	1.59	0.008	1.91	<0.001
Congenital anomalies of female genital organs	5.25	<0.001	4.46	<0.001
Early or threatened labor; hemorrhage in early pregnancy	1.44	<0.001	1.29	<0.001
Hemorrhage in early pregnancy	5.23	<0.001	6.49	<0.001
Hemorrhage (Not Otherwise Specified)	3.35	<0.001	2.65	<0.001
Miscarriage; stillbirth	2977.4	<0.001	1975.63	<0.001
Multiple gestation	1.56	<0.001	2.21	<0.001
Pituitary hyperfunction	3.74	<0.001	4.14	<0.001
Polyp of corpus uteri	4.09	<0.001	5.76	<0.001
Primary hypercoagulable state	4.2	<0.001	4.83	<0.001
Threatened premature labor	1.55	0.047	2.09	<0.001

**(b) Menstrual abnormalities**				

Absent or infrequent menstruation	1.67	<0.001	2.74	<0.001
Disorders of menstruation and other abnormal bleeding from female genital tract	2.27	<0.001	4.48	<0.001
Dysmenorrhea	1.96	<0.001	2.94	<0.001
Excessive or frequent menstruation	1.94	<0.001	2.97	<0.001
Irregular menstrual bleeding	1.8	<0.001	3.53	<0.001
Irregular menstrual cycle	2.16	<0.001	4.95	<0.001
Irregular menstrual cycle/bleeding	2.67	<0.001	4.41	<0.001

**(c) Infertility-associated diagnoses**				

Endometriosis	3.13	<0.001	2.73	<0.001
Infertility, female	5.07	<0.001	9.69	<0.001
Infertility, female, associated with anovulation	2.88	<0.001	5.1	<0.001
Ovarian dysfunction	3.17	<0.001	5.05	<0.001
Polycystic ovaries	2.15	<0.001	3.76	<0.001
Premature menopause and other ovarian failure	3.75	<0.001	3.4	<0.001

**(d) Infections & immunological conditions**				

Acute pharyngitis	1.44	0.004	1.65	<0.001
Inflammatory diseases of uterus, except cervix	1.96	0.009	10.48	<0.001
Other immunological findings	3.63	<0.001	4.61	<0.001
Pelvic inflammatory disease (Not Otherwise Specified)	4.29	<0.001	5.65	<0.001
Unspecified diffuse connective tissue disease	7.32	<0.001	2.56	<0.001
Vaginitis and vulvovaginitis	1.48	<0.001	1.97	<0.001

**(e) Other validated positive associations**				

Complications following abortion or ectopic and molar pregnancies	>1	<0.05	24.55	<0.001
Disorders of uterus (Not Elsewhere Classified)	2.26	<0.001	3.31	<0.001
Disorders secondary to childbirth, surgery, trauma	1.87	<0.001	2.26	<0.001
Disturbances of sulfur-bearing amino acid metabolism	3.52	<0.001	7.61	<0.001
Dysmetabolic syndrome X	9.56	<0.001	9.83	<0.001
Gastroparesis	3.3	0.034	>1	<0.05
Noninflammatory female genital disorders	1.76	<0.001	3.34	<0.001
Other symptoms	1.57	0.029	1.65	0.002
Ovarian cyst	2.12	<0.001	3.07	<0.001
Poisoning by analgesics, antipyretics, and antirheumatics	>1	<0.05	>1	<0.05

Several infertility-associated diagnoses are significantly positively associated with RPL. This includes both the broad “infertility” diagnosis (UCSF odds ratio: 5.07, Stanford odds ratio: 9.69) and more specific infertility-associated diagnoses such as endometriosis (UCSF odds ratio: 3.13, Stanford odds ratio: 2.73), polycystic ovaries/polycystic ovarian syndrome (PCOS) (UCSF odds ratio: 2.15, Stanford odds ratio: 3.76), ovarian dysfunction (UCSF odds ratio: 3.17, Stanford odds ratio: 5.05), and ovarian failure (UCSF odds ratio: 3.75, Stanford odds ratio: 3.4). All inter-center validated infertility associations have *p* < 0.001 from their respective GAMs.

Positive association results also include infections, immunological conditions, ovarian cyst, and dysmetabolic syndrome X (more commonly called “metabolic syndrome”). Inter-center validated negative association results primarily consist of diagnoses related to childbirth and pregnancy (Table 3). Discordant association results include diagnoses related to mental health, glucose, and pregnancy (Table 4).

**Table 3:** Negative associations

Diagnosis	UCSF odds ratio	UCSF *p*-value	Stanford odds ratio	Stanford *p*-value
Abnormality in fetal heart rate or rhythm	0.19	<0.001	0.25	<0.001
Abnormality of organs and soft tissues of pelvis complicating pregnancy, childbirth, or the puerperium	0.43	<0.001	0.44	<0.001
Acute posthemorrhagic anemia	0.41	<0.001	0.64	<0.001
Anemia during pregnancy	0.29	<0.001	0.51	<0.001
Carbuncle and furuncle	0.55	0.034	0.57	<0.001
Coagulation defects complicating pregnancy or postpartum	0.56	<0.001	0.65	<0.001
Complications of labor and delivery NEC	0.31	<0.001	0.47	<0.001
Diabetes or abnormal glucose tolerance complicating pregnancy	0.37	<0.001	0.43	<0.001
Fetal distress and abnormal forces of labor	0.26	<0.001	0.32	<0.001
Hypertension complicating pregnancy, childbirth, and the puerperium	0.28	<0.001	0.46	<0.001
Infectious and parasitic complications affecting pregnancy	0.34	<0.001	0.52	<0.001
Known or suspected fetal abnormality affecting management of mother	0.51	<0.001	0.67	<0.001
Late pregnancy and failed induction	0.22	<0.001	0.19	<0.001
Malposition and malpresentation of fetus or obstruction	0.37	<0.001	0.49	<0.001
Maternal complication of pregnancy affecting fetus or newborn	0.59	0.036	0.61	<0.001
Mental disorders during/after pregnancy	0.38	<0.001	0.63	<0.001
Normal delivery	0.14	<0.001	0.17	<0.001
Obstetrical/birth trauma	0.12	<0.001	0.12	<0.001
Other and unspecified complications of birth; puerperium affecting management of mother	0.38	<0.001	0.29	<0.001
Other anemias	0.42	<0.001	0.8	0.003
Other complications of pregnancy NEC	0.44	<0.001	0.75	<0.001
Other complications of the puerperium NEC	0.31	<0.001	0.55	<0.001
Other conditions or status of the mother complicating pregnancy, childbirth, or the puerperium	0.31	<0.001	0.44	<0.001
Other disorders of the breast associated with childbirth and disorders of lactation	0.38	<0.001	0.52	<0.001
Other perinatal conditions of fetus or newborn	0.85	0.008	0.76	<0.001
Other tests	0.38	<0.001	0.59	<0.001
Placenta previa and abruptio placenta	0.32	<0.001	0.51	<0.001
Preeclampsia and eclampsia	0.25	<0.001	0.39	<0.001
Problems associated with amniotic cavity and membranes	0.34	<0.001	0.44	<0.001
Retention of urine	0.44	<0.001	0.4	<0.001
Secondary thrombocytopenia	0.32	<0.001	0.39	<0.001
Thrombocytopenia	0.42	<0.001	0.61	<0.001
Umbilical cord complications during labor and delivery	0.26	<0.001	0.23	<0.001
Venous/cerebrovascular complications embolism in pregnancy and the puerperium	0.41	<0.001	0.54	<0.001

**Table 4:** Discordant associations

Diagnosis	UCSF odds ratio	UCSF *p*-value	Stanford odds ratio	Stanford *p*-value
Anxiety disorder	0.74	<0.001	1.4	<0.001
Constipation	0.76	0.025	1.29	0.004
Depression	0.7	0.034	1.78	<0.001
Generalized anxiety disorder	0.63	0.007	1.72	<0.001
Impaired fasting glucose	0.59	0.001	1.5	<0.001
Infections of genitourinary tract during pregnancy	0.66	<0.001	1.26	0.016
Iron deficiency anemias, unspecified or not due to blood loss	0.6	<0.001	1.32	0.003
Major depressive disorder	0.7	<0.001	1.57	<0.001
Other abnormal glucose	0.61	0.002	2.25	<0.001
Other high-risk pregnancy	0.7	<0.001	1.18	<0.001
Pruritus and related conditions	0.7	0.003	1.27	0.012
Rhesus isoimmunization in pregnancy	0.59	<0.001	1.51	0.003

### RPL-associated diagnoses have higher odds ratios for patients <35 years compared with patients 35+ years

Maternal age plays a key role in pregnancy loss risk. The risk starts increasing slightly around age 30 years and increases dramatically around age 35 years.^52^ Age is also an important factor for virtually every aspect of health. To compare RPL association patterns in younger vs. older patients, we implemented an age-stratified analysis with two age strata: <35 and 35+ years. We then formally tested whether associations differed between strata using two-sided z-tests of the coefficients. Demographics and healthcare utilization for <35 and 35+ patients at UCSF and Stanford are reported in Tables S2–S5.

**Figure 5:**
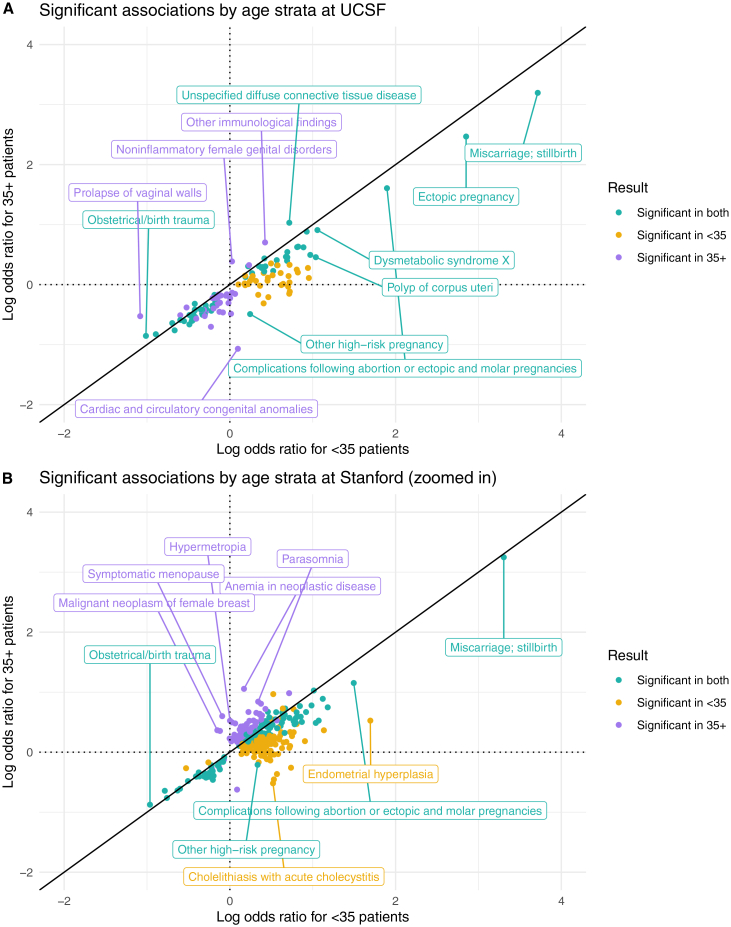
Age-stratified analysis results (A) At UCSF. (B) At Stanford (zoomed in for readability). The full Stanford plot with outliers is in Figure S7. All points in the log-log plots were filtered to include diagnoses where ≥10 patients in either the RPL or control group have a record of that diagnosis. All log transformations in these plots are in base 10.

In the UCSF age-stratified analysis, the associations of 1,419 candidate diagnoses with RPL were evaluated in both the <35 and 35+ strata (Data S2). To compare results between age strata, we computed the union of significant results from both the <35 and 35+ analyses. We found that 128 diagnoses are significant in at least one age stratum. While their odds ratios are ordinally correlated between strata (Spearman r = 0.889, *p*-value < 0.001), the vast majority (111/128) of odds ratios were higher in <35 patients compared with 35+ patients. Some examples include polyp of corpus uteri, metabolic syndrome, high-risk pregnancy, and complications following abortion or ectopic & molar pregnancies (Figure 5A). From the UCSF age-stratified coefficients z-tests, 14 diagnostic associations vary significantly between strata, including hemorrhage in early pregnancy and mental disorders during or after pregnancy.

In the Stanford age-stratified analysis, 1,512 diagnoses were tested in both the <35 and 35+ strata (Data S3). Of those, 342 are significant in at least one stratum. Their odds ratios are modestly correlated (Spearman r = 0.365, *p*-value < 0.001), and the majority (239/342) of odds ratios are higher in <35 patients compared with 35+ patients. Two examples are high-risk pregnancy and complications following abortion or ectopic & molar pregnancies (Figure 5B). From the Stanford age-stratified coefficients z-tests, 7 diagnostic associations varied significantly between strata, including hypothyroidism and noninflammatory disorders of the cervix.

### While Stanford results are sensitive to control for healthcare utilization, UCSF results are stable across analyses

In our healthcare utilization sensitivity analysis, we assessed whether controlling for healthcare utilization changed our association results. To do this, we re-estimated associations, adding a covariate for patients’ number of visits during the study window (any time before RPL/birth up until a year after RPL/birth). The number of visits was selected as a metric for healthcare utilization because it is a direct measure of how much contact a patient has had with the healthcare system. Then, we calculated the median percent difference between odds ratios without and with the number of visits included in the model.

**Figure 6:**
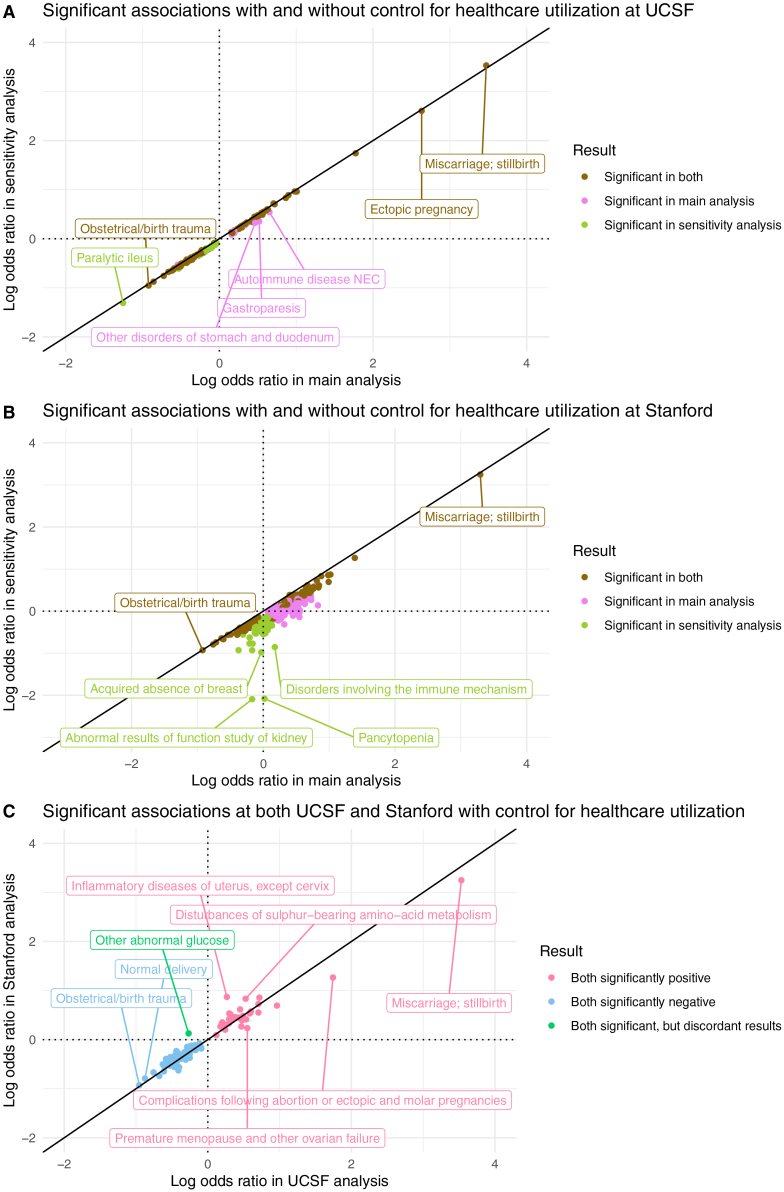
Healthcare utilization sensitivity analysis results (A) At UCSF. (B) At Stanford. (C) Validated positive, validated negative, and discordant association results, after accounting for healthcare utilization in both the UCSF and Stanford analyses. All points in the log-log plots were filtered to include diagnoses where ≥10 patients in either the RPL or control group have a record of that diagnosis. All log transformations in these plots are in base 10.

In the UCSF healthcare utilization sensitivity analysis, 138/1,612 diagnoses are significant: 42 positive and 96 negative. To compare association results between UCSF’s main analysis and UCSF’s sensitivity analysis, we computed the union of significant results from both.;148 diagnoses are significant in either analysis (Figure 6A). Their odds ratios are extremely highly correlated (Spearman r = 0.997, *p*-value < 0.001). After adjusting for healthcare utilization, odds ratios decrease modestly (median 13% decrease). Adjusted odds ratios and *p*-values from the UCSF sensitivity analysis are reported in Data S2.

In the Stanford healthcare utilization sensitivity analysis, 162/1,662 diagnoses are significant: 56 positive and 106 negative. Computing the union of significant results from Stanford’s main and sensitivity analysis yielded 421 diagnoses significant in either analysis (Figure 6B). Their odds ratios have very high ordinal correlation (Spearman r = 0.912, *p*-value < 0.001). However, the odds ratios’ values are quite different: after adjusting for healthcare utilization, odds ratios decrease substantially (median 49% decrease). Adjusted odds ratios and *p*-values from the Stanford sensitivity analysis are reported in Data S3.

In summary, healthcare utilization is associated with RPL, as evidenced by the reduction in significantly positive associations and their respective odds ratios, compared with the main models. These reductions are minor in the UCSF analysis and major in the Stanford analysis. However, significant results between the main and sensitivity analyses are highly correlated.

### Most of the inter-center validated results are sustained in models controlled for utilization

To ascertain which association results validated across medical centers after controlling for number of visits, we computed the intersection of significant results from both medical centers’ healthcare utilization sensitivity analyses (Figure 6C); 90 diagnoses are significant in both centers, and their odds ratios are very highly correlated (Spearman r = 0.944, *p*-value < 0.001). Of those 90 diagnoses, 33 diagnoses are positive in both, 56 are negative in both, and 1 is discordant (significant in both, but in opposite directions).

Next, we assessed whether the inter-center validated results from the main analysis were sustained in the models controlled for utilization. Of the 42 validated positive associations from the main analysis, 33 are also significantly positively associated with RPL at both UCSF and Stanford after controlling for utilization. All 34 validated negative associations from the main analysis are sustained in this sensitivity analysis.

### Subgroup analyses further support RPL association results

To investigate whether our results varied for patients with longer EHRs, we repeated our association analysis on the subgroup of RPL and control patients who have 10 or more years of EHR data prior to their index date (RPL date or live birth date). In the UCSF 10-year analysis, there are 686 RPL patients and 2,117 control patients. In the Stanford 10-year analysis, there are 614 RPL patients and 5,452 control patients. The results of our 10-year subgroup analysis are congruent with our main association analysis results. For example, irregular menstrual cycle, disorders of menstruation, infertility, endometriosis, ovarian dysfunction, and vaginitis/vulvovaginitis are all significantly positively associated with RPL at both UCSF and Stanford (Data S8 and S9).

To examine diagnostic associations for severe RPL, we implemented a subgroup analysis of RPL patients with three or more pregnancy losses at UCSF or Stanford. The UCSF severe RPL analysis includes 321 RPL and 17,259 control patients. The Stanford severe RPL analysis includes 354 RPL and 36,019 control patients. Severe RPL association results are consistent with the main RPL association analysis, including irregular menstrual cycle/bleeding, excessive menstruation, absent menstruation, infertility, PCOS, and vaginitis/vulvovaginitis (Data S10 and S11).

### Life after loss: Predictors of live birth among RPL patients

We conducted an exploratory analysis of whether the identified associations from the main analysis are predictive of RPL patients’ next pregnancy outcomes. Of the 3,840 patients in the UCSF RPL group, 1,493 have a live birth or pregnancy loss recorded at UCSF after their RPL index date. This includes 759 births and 734 losses. For each of the 88 RPL-associated diagnoses from the main analysis, we determined whether it was present in patients’ EHRs before their next pregnancy outcome and estimated its association with birth vs. loss using a confounder-adjusted GAM (Data S12).

Overall, the odds ratios are significantly correlated between analyses (Spearman r = 0.785, *p*-value < 0.001); 48 diagnoses are significantly negatively associated with subsequent loss (positively associated with subsequent birth) and 1 diagnosis is significantly positively associated with subsequent loss. For 41 of the significant associations, the directionality of the association is concordant between the next pregnancy analysis and the main RPL analysis at UCSF; 8 of the significant associations have discordant directionality between the two analyses.

Diagnoses that are significantly negatively associated with loss (positively associated with birth) across both analyses include many late pregnancy or delivery-related complications, such as thrombocytopenia (odds ratio for loss: 0.317, *p*-value: 0.004), malposition and malpresentation of fetus or obstruction (odds ratio for loss: 0.21, *p*-value < 0.001), and problems associated with amniotic cavity and membranes (odds ratio for loss: 0.22, *p*-value < 0.001). Metabolic syndrome is positively associated with loss (odds ratio for loss>1, *p*-value < 0.05) in the next pregnancy analysis, which is consistent with findings in the main analysis. However, one of the comparator groups for metabolic syndrome had <10 patient count so the exact odds ratio and *p*-value are redacted as part of best practices for maintaining EHR data deidentification and protecting patient privacy.

## Discussion

We conducted a large-scale association analysis to identify diagnoses positively or negatively associated with RPL in two independent EHR databases. Additionally, we compared RPL associations in younger vs. older patients using an age-stratified analysis and assessed potential confounding from healthcare utilization using a sensitivity analysis with the number of visits. Our positive association results replicate several known RPL associations spanning chromosomal, anatomical, endocrine, and coagulation conditions (Table 2a). We also report several potentially novel and clinically intriguing positive associations, characterized below.

A constellation of menstrual abnormalities are positively associated with RPL, ranging from absent/infrequent menstruation to excessive/frequent menstruation (Table 2b). Menstrual abnormalities could be important risk factors for RPL. They are easily screened and monitored over time, especially with the rise of menstrual health apps.^53^ Additionally, there are several potential mechanisms for menstrual abnormalities to be associated with pregnancy loss. They could signal inadequate progesterone production,^54^ which could be caused by thyroid or ovarian dysfunction.^54^^,^^55^^,^^56^^,^^57^ There are also structural causes of menstrual abnormalities, including fibroids, polyps, or other uterine anomalies.^58^^,^^59^^,^^60^ There is considerable variation in the formal^61^^,^^62^ and in-practice^63^^,^^64^ guidelines for RPL evaluation and management. Depending on the clinical workup that RPL patients receive, some of them may have undiagnosed hormonal or structural abnormalities but present as abnormal menses.

Two smaller studies on menstrual abnormalities and pregnancy loss reported positive associations.^65^^,^^66^ However, both of their association results were non-significant, possibly due to being under-powered. The first study (*N* = 2,046) found a non-significant positive association between short (≤25 days) menstrual cycle length and pregnancy loss.^65^ The second study (*N* = 252) reported that individuals with short (<10 days) luteal phases in all three pre-conception menstrual cycles had a non-significant increased risk of pregnancy loss.^66^ Our study included a large patient population (*N* = 61,774), and our reported menstrual abnormality results are statistically significant (all *p*-values < 0.001 from their respective GAMs). Compared with prior studies, our study has a larger number of patients, different choice of confounder adjustment, and different study population. Future studies on additional populations can further elucidate the complex relationship between menstrual health and pregnancy outcomes.

In our study, both infertility and infertility-associated diagnoses (e.g., endometriosis, PCOS, ovarian dysfunction/failure) are positively associated with RPL (Table 2c). While RPL is the inability of pregnancies to reach viability, infertility is the inability to conceive a pregnancy. There is long-standing debate about whether RPL and infertility are connected.^67^^,^^68^^,^^69^^,^^70^ Infertility and pregnancy loss may be related as implantation disorders could affect both the ability to conceive and establish a healthy ongoing pregnancy.^71^ For instance, progesterone resistance could cause menstrual abnormalities, also leading to infertility and pregnancy loss.^72^ Alternatively, an individual might have a genetic predisposition to abnormal oogenesis, which could also cause infertility and RPL.^73^ It is now recognized that numerous genes involved in responding to DNA damage play a pivotal role in causing infertility.^74^ Therefore, it is possible that infertility, especially when associated with variants of DNA damage response genes, may increase the likelihood of aneuploidy. This anomaly can subsequently lead to conceptions characterized by genome instability and aneuploidy, resulting in pregnancy loss. Another possibility is that menstrual irregularities due to ovarian dysfunction and subfertility could be caused by these genes. However, these mechanistic hypotheses are speculative in nature; there is a potential for a diagnostic bias and a mediation analysis with molecular data could formally evaluate the hypotheses presented here.

A previous RPL EHR study of UKBB data also reported a positive association between RPL and infertility.^24^ Although, in EHR data, the association between the broad “infertility” diagnosis and RPL may be affected by selection bias or confounding bias. Selection bias can result from the fact that these patients might be undergoing *in vitro* fertilization treatment. Infertility patients may be more likely to seek medical help regarding conception and viability of their pregnancies. Confounding bias could arise from RPL patients incorrectly receiving an infertility diagnosis instead of an RPL diagnosis. For example, pregnancies may be lost before they are recognized, leading to inaccurate infertility diagnoses.^67^

Several prior studies have investigated potential associations between RPL and infertility-associated diagnoses. Our endometriosis findings are consistent with a recent nationwide cohort study in Denmark that found a significant positive association between endometriosis and pregnancy loss, including stronger associations with increasing number of losses.^75^ Prior literature on a relationship between PCOS and RPL is mixed, with a couple of studies reporting significant positive associations^30^^,^^76^ and another reporting no significant association.^77^ Our PCOS results provide further evidence of a positive association between PCOS and RPL. There is limited previous literature on an association between ovarian failure/dysfunction and pregnancy loss, likely due to low conception rates within those patients. A 1999 systematic review found that primary ovarian failure patients had a pregnancy loss rate comparable with the general population.^78^ However, in our study, we observe a significant positive association with RPL for both ovarian dysfunction and ovarian failure. There are a couple potential mechanisms for this association. For individuals with diminished ovarian reserves, their remaining eggs may be less likely to be euploid,^79^ which could contribute to aneuploidy-related RPL.^80^ Additionally, hormonal production dysregulation in individuals with ovarian dysfunction/failure could contribute to pregnancy loss risk.

Our results for vaginitis/vulvovaginitis and pelvic inflammatory disease (Table 2d) suggest that the vaginal and uterine microbiome could play a role in pregnancy loss risk. While vaginal microbiome composition has been widely studied in the context of preterm birth,^81^^,^^82^^,^^83^^,^^84^ its role in pregnancy loss is less understood. Two previous studies reported that vaginal microbiome composition was associated with both pregnancy loss^85^ and history of pregnancy loss.^86^ History of pregnancy loss has also been associated with bacterial vaginosis and vulvovaginal candidiasis.^87^ Additionally, vaginal microbial dysbiosis was recently reported to be associated with euploid pregnancy loss.^88^ Lastly, there is evidence that untreated chronic endometritis can contribute to pregnancy loss risk.^89^^,^^90^^,^^91^ In aggregate, our results and those of previous studies indicate that the vaginal and uterine microbiome may be important for unraveling RPL etiology. These could also represent potential targets for the development of new treatments for RPL.

Metabolic syndrome is very positively associated with RPL at both UCSF and Stanford (Table 2e). A relationship between these two conditions has been previously reported,^92^ with one study hypothesizing that metabolic syndrome mediates inflammatory and oxidative stress responses in RPL.^93^ However, another study did not find any significant associations between metabolic parameters and RPL.^94^ Additionally, metabolic syndrome is very common in patients with PCOS,^95^ which has also been associated with RPL. Further studies are needed to discern whether PCOS is confounding the association between metabolic syndrome and RPL, or whether there is a causal link between metabolic syndrome and RPL. Given the magnitude of the association between metabolic syndrome and RPL observed at both UCSF and Stanford, investigating the impact of metabolic health interventions on RPL patients’ subsequent pregnancy outcomes could be quite clinically relevant.

Our positive association results also include miscarriage, stillbirth, ectopic pregnancy, and complications following abortion or ectopic and molar pregnancies. These associations are driven by how the case and control groups were defined: pregnancy losses in the case group and no pregnancy losses in the control group. The negative association results in our study are mainly related to childbirth/pregnancy (Table 3). Most of these conditions occur in the second trimester, so they are presumably from pregnant individuals in the control group. The replication of known associations with pregnancy and live birth are further affirming of our overall approach.

There are a handful of diagnoses with discordant results. While their *p*-values are significant at both UCSF and Stanford, their odds ratios are in opposite directions—they are negatively associated with RPL at UCSF and positively associated with RPL at Stanford. Discordant results include anxiety, depression, impaired glucose, and abnormal glucose (Table 4). These discordances are most likely due to differences in screening practices between the two medical centers, which should be explored further.

Across both UCSF and Stanford, 35+ patients have longer EHRs, more visits, and more diagnoses compared with <35 patients (Tables S2–S5). In our age-stratified analysis, odds ratios tend to be higher in <35 patients vs. 35+ patients (Figure 5). One potential explanation for this result is that age plays an increasing role in pregnancy loss risk the older that patients get,^52^ so this could lead to other factors playing a decreasing role as patients age. This result could also be explained by the older population simply having more diagnoses overall. The differences in odds ratios between age strata are less pronounced at Stanford (Figure 5B) compared with UCSF (Figure 5A). Additionally, in the UMAP visualizations, there is less separation between age strata at Stanford (Figure 3D) than at UCSF (Figure 3B). Future studies could investigate the nature of age-related patterns for RPL-associated diagnoses, including testing for effect modifications or interactions.

Controlling for healthcare utilization as measured with visit count reduces both the proportion of significantly positive associations and the odds ratios for significant diagnoses. These reductions are small at UCSF (Figure 6A) but larger at Stanford (Figure 6B). This is likely because the difference in utilization levels between RPL and controls is much more pronounced at Stanford than at UCSF. Stanford RPL patients have many more visits than Stanford control patients (median: 31 vs. 14) (Table S1). However, UCSF RPL patients have a similar number of visits as UCSF control patients (median 42.5 vs. 41) (Table 1). The site-specific differences may be driven by differences in patients’ care-seeking behaviors, differences in the analytic samples in this study, or a combination of clinical- and data-related differences.

We controlled for healthcare utilization in sensitivity analyses rather than primary results because utilization can be either a cause or a consequence of many of the candidate diagnoses we evaluated. Without greater temporal resolution, it is therefore unclear whether analyses should be adjusted for utilization. For example, patients with higher utilization may be more likely to receive an RPL diagnosis and any other diagnoses.^96^ To address this confounding bias, we can adjust for utilization by adding it as a covariate in our models. However, including utilization in our models could *lead* to an underestimation of the association between each candidate diagnosis and RPL (i.e., collider bias), because having diagnoses such as menstrual abnormalities, infertility, or PCOS may increase healthcare utilization. Thus, it is useful to compare models with and without utilization to assess potential confounding bias, but utilization was not included in our main model to avoid introducing collider bias.

These findings demonstrate that healthcare utilization can play a minor role in some studies (e.g., UCSF analysis) and a major role in other studies (e.g., Stanford analysis), so it must always be evaluated carefully. The dramatic change in Stanford results after controlling for utilization illustrates how sensitive effect size estimates can be in EHR association analyses when the comparison groups have very different utilization levels. However, it is important to note that the changes in effect estimates should not be interpreted as exact measures of confounding. Rather, these changes reflect a combination of confounding and noncollapsibility.^97^

Overall, it is affirming to observe that most of the inter-center validated results are sustained in models controlled for utilization. This illustrates the utility of external validation for identifying associations that are robust across center-specific healthcare utilization patterns. Moreover, it is affirming that the association results are stable in the UCSF data when comparing between the main vs. sensitivity analysis (Figure 6A). This suggests that healthcare utilization is not a major confounder or collider in the UCSF analyses.

A large and diverse patient population is included in our study (Tables 1 and S1). We observed significant differences in the distributions of patients’ identified races and ethnicities between RPL and control groups at both UCSF and Stanford. In particular, fewer RPL patients were identified as Hispanic or Latino compared with control patients (UCSF: 11.7% vs. 16.6%, Stanford: 16.4% vs. 29.3%). Future studies could further examine these differences, with the goals of investigating sources of selection bias in the underlying datasets, assessing potential confounders, and if applicable, identifying interventions to reduce any disparities in pregnancy loss risk and clinical care.^98^^,^^99^

Our study has several strengths: this is the first RPL clinical association study to focus on diagnoses occurring before and near RPL onset, with the goal of generating hypotheses about RPL etiologies. We included a large patient population, with 8,496 RPL and 53,278 control patients. From a methodological perspective, we externally validated our results in a separate EHR database, and we assessed the sensitivity of those results to healthcare utilization. Lastly, we provide a framework including phenotype definitions (Data S1), which can be used by other researchers to study RPL using EHR data.

In total, our association analysis identified 48 diagnoses that are significantly positively associated with RPL at both UCSF and Stanford. This includes several menstrual abnormalities, spanning from absent/infrequent menstruation to excessive/frequent menstruation. Prior studies with smaller sample sizes found similar menstrual associations, but their results did not reach statistical significance. We also see a strong positive association with infertility—both the broad “infertility” diagnosis and more specific infertility-associated diagnoses including endometriosis, PCOS, and ovarian failure/dysfunction. This adds additional weight to the long-standing but contentious hypothesis that RPL and infertility could be connected. Subsequent studies can dive more deeply into the hypothesized biological mechanisms for these associations, explore the possibility of latent factors affecting the observed relationship, evaluate their prognostic value for RPL patients’ future pregnancy outcomes, and explore whether the reported diagnostic associations could inform therapeutic strategies to help RPL patients.

### Limitations of the study

There are also limitations to our study. The pregnancy loss concepts in the EHR generally do not specify the gestational age that the pregnancy was lost, so we cannot incorporate that into our analyses. Excluding patients with prior losses from the control group may have led to an exaggerated difference in age distributions between control and RPL patients. Although we filtered control patients for no history of pregnancy loss, it is possible that some control patients had previous pregnancy losses that were not recorded in EHR data, leading to misclassification of case/control status. While we are able to account for a number of covariates, there are some confounders that are not included because they are not captured in the EHR. Integrating with additional datasets such as those capturing social determinants of health measures and imaging data would be valuable as follow-up studies. More broadly, patients’ reproductive periods in the EHR databases are often subsets of their lifetime reproductive periods, so the prevalence of RPL (and patients with live births) will inevitably not match population-level prevalence estimates. For the next pregnancy analysis, we focused on two outcomes: pregnancy loss or live birth. This analysis did not take additional outcomes into consideration, such as terminations. Across all of the analyses, it is important to note that correlation does not necessarily indicate causation. Some associations may have arisen by chance owing to the large number of comparisons we made, confounding given that we could not control for numerous potential social and behavioral risk factors, or incidentally during pregnancy, fertility workups, or RPL workups. To directly address this, future studies would need to evaluate the unique data provenance of each diagnosis.^100^ Also, some diagnostic records may be inaccurate, either due to clinician miscodings or conversion errors propagated throughout the deidentification or standardization processes. Another limitation is that both UCSF and Stanford are academic medical centers in a similar region of California. It is possible that some patients received care at both UCSF and Stanford, and patients in these medical centers are not necessarily representative of pregnant individuals in the general population. The percentage of RPL patients with previous live births at our medical center is lower than population-level prevalence of secondary RPL.^50^^,^^101^^,^^102^^,^^103^ Similarly, the percentage of RPL patients who go on to have another pregnancy at our medical center may be lower than expected. This finding is not surprising or unusual for an EHR study and is sometimes referred to as informative presence bias. There is a chance that we are not capturing all pregnancies in this study and patients could have pregnancies outside the UCSF health system. Patients move in and out of health care systems; even if someone stays in the Bay Area they may move closer to another hospital and continue care there or go back to their primary care providers. For example, UCSF provides a lot of tertiary and quaternary care, so patients seeking primary care may be underrepresented at UCSF. While our inter-center validation demonstrates some generalizability, further work is needed to assess generalizability in other populations.^104^^,^^105^

## Resource availability

### Lead contact

Further information and requests for resources and reagents should be directed to and will be fulfilled by the lead contact, Marina Sirota (marina.sirota@ucsf.edu).

### Materials availability

This study is purely computational and did not generate new unique reagents.

### Data and code availability

All EHR concept lists are in Data S1. UCSF and Stanford diagnosis association results are in Data S2, S3, S4, S5, S6, S7, S8, S9, S10, and S11. In our association analyses, some diagnoses had very low (<10) patient counts. To maintain patient deidentification, exact counts, odds ratios, and *p*-values are redacted for those diagnoses. UCSF-affiliated individuals can request access to UCSF EHR data by contacting UCSF Information Commons (info.commons@ucsf.edu). Stanford’s EHR data are managed through the Stanford Research Repository (https://med.stanford.edu/starr-tools.html). Individuals not affiliated with UCSF may request to set up an official collaboration with a UCSF-affiliated investigator by contacting the principal investigator, Marina Sirota (marina.sirota@ucsf.edu). Requests should be processed within a couple of weeks.

Our GitHub repository (https://github.com/jackieroger/RPL_association_study) contains instructions for OMOP EHR data queries and all of the code for patient filtering, diagnosis aggregation, main association analysis, age-stratified analysis, healthcare utilization sensitivity analysis, 10-year subgroup analysis, severe RPL subgroup analysis, comparing results, and creating figures.

## Acknowledgments

Thank you to Silvia Miramontes, Umair Khan, and Ron Wong for reviewing and editing our manuscript. Thank you to Evan Phelps for answering questions about clinical data mapping, aggregation, and deidentification. Thank you to Tony Capra for answering statistical questions. Thank you to Tim Wen for clinical perspective. The authors acknowledge the use of resources developed and supported by the 10.13039/100005544UCSF Bakar Computational Health Sciences Institute Information Commons team and thank members of this team for technical support. This material is based upon work supported by the 10.13039/100023581National Science Foundation Graduate Research Fellowship Program under Grant No. 2038436 (J.R.). We would also like to acknowledge the following funding sources: T32GM067547 (J.R.), R01HD105256 (M.S., A.R., R.B.L., M.P.S., N.A.), March of Dimes (T.T.O, G.M.S., D.K.S., N.A., M.S.), T32GM007618 (A.S.T.), R35GM138353 (N.A.), 1R01HL139844 (N.A.), 3P30AG066515 (N.A.), 1R61NS114926 (N.A.), 1R01AG058417 (N.A.), P01HD106414 (N.A.), the 10.13039/100000861Burroughs Welcome Fund (N.A.), 10.13039/100000968American Heart Association 19PABHI34580007 (N.A.), the 10.13039/100013829Alfred E. Mann Foundation (N.A.), the 10.13039/100013961Robertson foundation (N.A.), and the 10.13039/100015521Stanford Maternal and Child Health Research Institute through the Postdoctoral Support Award Program (F.X.). This research used data in the November 2022 release of the UCSF deidentified OMOP EHR database from the UCSF Academic Research Systems. It was supported by the 10.13039/100006108National Center for Advancing Translational Sciences, 10.13039/100000002National Institutes of Health, through UCSF-CTSI Grant UL1TR001872. This research used data or services provided by the 10.13039/100006056Stanford medicine Research data Repository (STARR), a clinical data warehouse containing live EPIC data from 10.13039/100006057Stanford Health Care (SHC), the Stanford Children’s Hospital (SCH), the University Healthcare Alliance (UHA), and Packard Children’s Health Alliance (PCHA) clinics and other auxiliary data from hospital applications such as radiology PACS. STARR platform is developed and operated by the Stanford Medicine Research IT team and is made possible by the Stanford School of Medicine Research Office. Any opinions, findings, and conclusions or recommendations expressed in this material are those of the authors and do not necessarily reflect the views of the National Science Foundation or the National Institutes of Health.

## Author contributions

J.R., M.S., R.B.L., H.C., M.M.G., and A.S.T. designed the study. J.R., M.S., M.M.G., G.M.S., D.T., and J.C. formulated the statistical analyses. J.R., A.S.T., T.T.O., S.R.W., I.K., and B.L.L. navigated UCSF EHR data access and Phecodes diagnosis aggregation. J.L. developed the initial strategy for identifying transgender, non-binary, and gender-diverse individuals in EHR data, and J.R. applied that strategy for the OMOP data framework. J.R., M.S., F.X., and N.A. determined how to port the analysis models from UCSF to Stanford. J.R. carried out the UCSF analyses, and F.X. carried out the Stanford analyses. R.B.L., H.C., A.R., D.K.S., L.C.G., and M.P.S. shared clinical and biological insights for interpreting the results. J.R. and F.X. created the figures. J.R. wrote the manuscript. J.R., M.S., M.M.G., T.T.O., G.M.S., A.R., R.B.L., S.R.W., D.K.S., D.T., J.M.C., F.X., N.A., A.S.T., and B.L.L. edited the manuscript. All authors read and approved the final manuscript.

## Declaration of interests

J.R. interned at Roche and is employed by Kaiser Permanente. M.P.S. is a cofounder and scientific advisor of Personalis, SensOmics, Qbio, January AI, Fodsel, Filtricine, Protos, RTHM, Iollo, Marble Therapeutics, Crosshair Therapeutics, and Mirvie. M.P.S. is a scientific advisor of Jupiter, Neuvivo, Swaza, and Mitrix. N.A. is a member of the Scientific Advisory Boards of January AI, Parallel Bio, and WellSim Biomedical Technologies and is a paid consultant for MaraBio Systems. R.B.L. is on an advisory board for BioRad. D.T. is a paid consultant for Invitae Corp. L.C.G. is a consultant for BioRad, Sumitomo Pharma, Celmatix, NextGen Jane, ReproBio, Gesynta Pharmaceuticals, and Chugai Pharmaceuticals. M.S. is an advisor to Wellcome Leap.

## STAR★Methods

### Key resources table

**Table:** 

REAGENT or RESOURCE	SOURCE	IDENTIFIER
**Software and algorithms**		

Code	GitHub repository	https://github.com/jackieroger/RPL_association_study

### Experimental model and study participant details

No experimental models were leveraged in this analysis.

#### Patient selection

This study is reported in accordance with the Strengthening the Reporting of Observational Studies in Epidemiology (STROBE) reporting guidelines. To identify which patients had records of RPL, live birth, or adverse pregnancy outcomes, we curated lists of Observational Medical Outcomes Partnership (OMOP) concepts for each pregnancy outcome. Curation was based on string-matching with search terms, and then manual review with guidance from clinical experts. Each OMOP concept list is included in Data S1. This can be used as a reference in other OMOP-based studies of RPL.

The entire UCSF patient selection process is summarized in Figure 2. UCSF patients were selected from the UCSF OMOP deidentified EHR database. In the deidentified database, patients’ names, addresses, and other identifying information are removed. Their absolute timelines are shifted, however relative timelines (i.e. the number of days between visits) are maintained. UCSF OMOP contains data from 6,400,834 patients spanning 1982-2022.

Patients were included in the RPL group if they had an RPL diagnosis or at least two pregnancy losses. Patients could fulfill the latter criterion by either (1) having a pregnancy loss after a recorded “history of pregnancy loss” or (2) having two pregnancy losses recorded at least 90 days apart from each other. The 90 day cutoff was chosen to ensure that the pregnancy loss records referred to two separate pregnancy losses, and not multiple records for the same loss. The full list of pregnancy loss concepts is included in Data S1, and includes chemical pregnancy, miscarriage, fetal death, and stillbirth. Molar pregnancies and extrauterine pregnancies were not explicitly included or excluded in our pregnancy loss queries. When selecting RPL patients, we imposed no restrictions on consecutiveness of pregnancy losses or any history of previous live births. For RPL patients with more than two pregnancy losses or multiple RPL diagnoses on different dates, we assigned their RPL index date as the earliest date that they met any of the RPL inclusion criteria.

Patients were included in the control group if they had any record of a diagnosis of uncomplicated live birth, normal delivery, or full-term live birth. The full list of live birth concepts is included in Data S1. Patients were excluded from the control group if they had any record of: pregnancy loss, preterm birth, preterm labor, preterm rupture of membranes, multiple gestation with loss, molar pregnancy, or extrauterine pregnancy. For control patients with more than one live birth, we assigned their live birth index date as the date of their first live birth.

For all included patients, their age was calculated by subtracting their birthdate from their RPL date (for RPL patients) or live birth date (for control patients). Then, all RPL and control patients were subjected to the following exclusion criteria: aged less than 15 or greater than 44 years at RPL onset (or live birth for control patients), neither female nor any record of incident pregnancy, total EHR length 100 or more years (which seemed biologically unlikely), or no diagnoses in the study period.

### Method details

#### Ethical approval

This study was approved by the Institutional Review Board of University of California San Francisco (#17-22929) and by the Institutional Review Board of Stanford University (#39225).

#### Diagnosis querying and aggregation

Patients’ ICD-based diagnostic histories were queried. Any ICD9, ICD9-CM, ICD10, or ICD10-CM diagnosis occurring before RPL onset (or before first live birth for control patients) up until a year afterwards was included. All diagnoses were then aggregated using Phecodes version 1.2.^106^ Phecodes (https://phewascatalog.org/phecodes) provides a crosswalk for assembling ICD9 and ICD10 diagnoses into clinically-relevant phenotypes. There are two main advantages to this: (1) Analogous ICD9 and ICD10 diagnoses can be tested together, instead of arbitrarily separately. (2) The ICD hierarchy has many levels of specificity, and Phecodes aggregation allows us to group together similar diagnoses that are unnecessarily specific for our analysis.

#### Visualizing patients’ EHR patterns with UMAP

To visualize overarching diagnosis patterns in patients, we applied UMAP to all non-pregnancy-related diagnoses. First, we created a dataframe where each row was a patient and each column was a diagnosis. All diagnoses occurring in at least 1 RPL or control patient were included. For each patient, the presence or absence of each diagnosis in their records was one-hot-encoded. Next, we filtered out any diagnoses in the Phecodes category “pregnancy complications”. This was to ensure that the subsequent dimensionality reduction was based on diagnoses leading to the outcome (RPL or live birth), instead of diagnoses indicating the outcome. We applied UMAP to the dataframe using the R package umap.^107^ This reduced the dimensionality of the diagnosis data into two dimensions. We visualized the resulting UMAP coordinates using the R package ggplot2,^108^ where points were colored based on whether the patient was in the RPL or control group. We tested whether the UMAP coordinate distributions were significantly different between RPL and control patients using wilcoxon rank sum tests. The visualization coloring and coordinate distribution testing was also carried out for comparing patients <35 vs patients 35+. Additionally, we visualized UMAP coordinates across patients’ race, ethnicity, number of visits, years in EHR, and number of diagnoses (Figures S2–S5).

### Quantification and statistical analysis

#### Association analysis

For all diagnoses occurring in at least one RPL or control patient, we implemented a case-control study to test the associations between each diagnosis and RPL. Crude associations were computed using logistic regression models. We used the glm() function in base R.^109^ Confounder-adjusted associations were computed using GAMs. We used the gam() function in the R package mgcv.^110^ The model covariates were maternal age, race, and ethnicity. A smoothing spline was applied to age to capture the non-linear relationship between age and pregnancy loss.^52^ Race and ethnicity were included to mitigate potential confounding from social determinants of health that may be correlated with race or ethnicity, including the likelihood that patients receive RPL (or additional) diagnoses. All *p*-values were adjusted for multiple testing using the Benjamini-Hochberg method. Association results were visualized with a manhattan plot and a volcano plot, using the R package ggplot2.^108^

#### Age-stratified analysis

Given the significant role that age plays in pregnancy loss^52^ and in health broadly, we hypothesized that RPL association patterns may vary across age strata. We compared RPL associations in younger vs older patients using an age-stratified analysis. To do this, we separated patients into two strata based on whether they were <35 or 35+ at the first record of RPL onset (or first live birth for control patients). Then, we ran two separate association analyses for <35 and 35+ patients. Results were visualized with a log-log plot using the R package ggplot2.^108^ Lastly, we formally tested whether association results varied across age strata using two-sided z-tests of the coefficients, and adjusted for multiple testing using the benjamini-hochberg method.

#### Healthcare utilization sensitivity analysis

We hypothesized that healthcare utilization could confound our association results. To investigate this, we re-ran our association analysis with the number of visits (in the study window) included as a covariate, in addition to the previously included covariates. For each diagnosis, we calculated the percent difference of its adjusted odds ratio before and after including visit number in the model. To summarize changes across all candidate diagnoses, we computed the median of these percent differences. Results were visualized with a log-log plot using the R package ggplot2.^108^

#### Confidence intervals for odds ratios

Across the main, age-stratified, and utilization analyses, we calculated 95% confidence intervals for the covariate-adjusted odds ratios to provide an additional opportunity for interpreting statistical significance of the association results.

#### 10-year subgroup analysis

To examine whether EHR length impacted association results, we conducted a sensitivity analysis restricted to patients with at least 10 years of data prior to patients’ index date (first live birth for control patients and first record of RPL for RPL patients). For those patients, we carried out a diagnosis association analysis, and then compared the results with the results from the main association analysis.

#### Severe RPL subgroup analysis

To investigate diagnostic associations among patients with more severe RPL, we carried out an association analysis where the case group was restricted to RPL patients with three or more recorded losses at UCSF. To ensure we were capturing distinct pregnancy losses instead of multiple records per loss, we only counted pregnancy loss records that occurred at least 90 days after the start of the previous recorded loss. For patients in the severe RPL subgroup, their index date was defined as the earliest date that they had records of at least three separate losses. The control patients’ index date was defined as the date of their earliest record of full-term live birth. The same control patients were used in the severe RPL subgroup analysis as were used in the main analysis.

#### External validation using the Stanford EHR database

We queried the Stanford OMOP deidentified EHR database containing 3,604,034 patients spanning 1994-2022.^111^ Patients were selected using the same criteria as described for the UCSF patients. The number of Stanford patients remaining at each step of the selection process is summarized in Figure S1. All three association analyses (main, age-stratified, healthcare utilization) were repeated on the Stanford data. Stanford results were compared to UCSF results by (1) quantifying overlaps between significant positive and negative associations, (2) computing correlations between odds ratios, and (3) visualizing results with Log-Log plots using the R package ggplot2.^108^ Subgroup analyses (10-year and severe RPL) were also repeated on the Stanford data.

#### Next pregnancy analysis

To explore whether the identified RPL-associated diagnoses influence next pregnancy outcomes, we analyzed subsequent births and losses of RPL patients. Among the patients in the RPL group, we identified births by linking between the UCSF OMOP database and the UCSF Clinical Data Warehouse (CDW) database and querying for deliveries in CDW. Losses were identified in the OMOP data using the pregnancy loss concept list included in Data S1. Any RPL patient with a recorded birth or loss at least 30 days after their RPL index date was included in this analysis. For RPL patients with multiple subsequent outcomes recorded, we restricted our analysis to their first one.

For each of the RPL-associated diagnoses reported in the main analysis, we estimated their association with birth vs loss in the next pregnancies using GAMs adjusted for maternal age, race, and ethnicity. For each patient in this analysis, a diagnosis was considered present in their EHR if there was any record of it before the date of their next pregnancy outcome (birth or loss). If not, it was considered not present in their record for this association analysis. All *p*-values were adjusted for multiple testing using the Benjamini-Hochberg method.

## Supplemental information

Document S1. Figures S1–S7, Tables S1–S5, and Methods S1

Data S1. Concept lists for diagnoses in the inclusion and exclusion criteria

Data S2. Association analysis results for UCSF

Data S3. Association analysis results for Stanford

Data S4. Confidence intervals for UCSF association analysis results

Data S5. Confidence intervals for Stanford association analysis results

Data S6. Coefficient z-tests for UCSF age-stratified analysis

Data S7. Coefficient z-tests for Stanford age-stratified analysis

Data S8. 10-year analysis results for UCSF

Data S9. 10-year analysis results for Stanford

Data S10. Severe RPL subgroup analysis results for UCSF

Data S11. Severe RPL subgroup analysis results for Stanford

Data S12. Next pregnancy analysis results

Data S13. Time window sensitivity analysis results

Data S14. Prior live birth sensitivity analysis results

Data S15. Propensity score matching sensitivity analysis results

Data S16. Infertility sensitivity analysis results

Data S17. Results from sensitivity analysis of <35 patients adjusted for healthcare utilization
