# Supplementary material for: Leveraging health records to identify diagnoses associated with recurrent pregnancy loss across two medical centers
Source: iScience. 2026 Jan 7;29(2):114633. doi: 10.1016/j.isci.2026.114633 (PMC12877842; doi:10.1016/j.isci.2026.114633)
Supplement: Document S1. Figures S1–S7, Tables S1–S5, and Methods S1 [file mmc1.pdf]

iScience, Volume *29*

Supplemental information

Leveraging health records to identify diagnoses associated with recurrent pregnancy loss across two medical centers

Jacquelyn Roger, Feng Xie, Jean M. Costello, Alice S. Tang, Jay Liu, Tomiko T. Oskotsky, Sarah R. Woldemariam, Idit Kosti, Brian L. Le, Michael P. Snyder, Linda C. Giudice, Gary M. Shaw, David K. Stevenson, Aleksandar Rajkovic, M. Maria Glymour, Dara Torgerson, Nima Aghaeepour, Hakan Cakmak, Ruth B. Lathi, and Marina Sirota

Supplementary figure 1: Inclusion and exclusion criteria for Stanford patients.

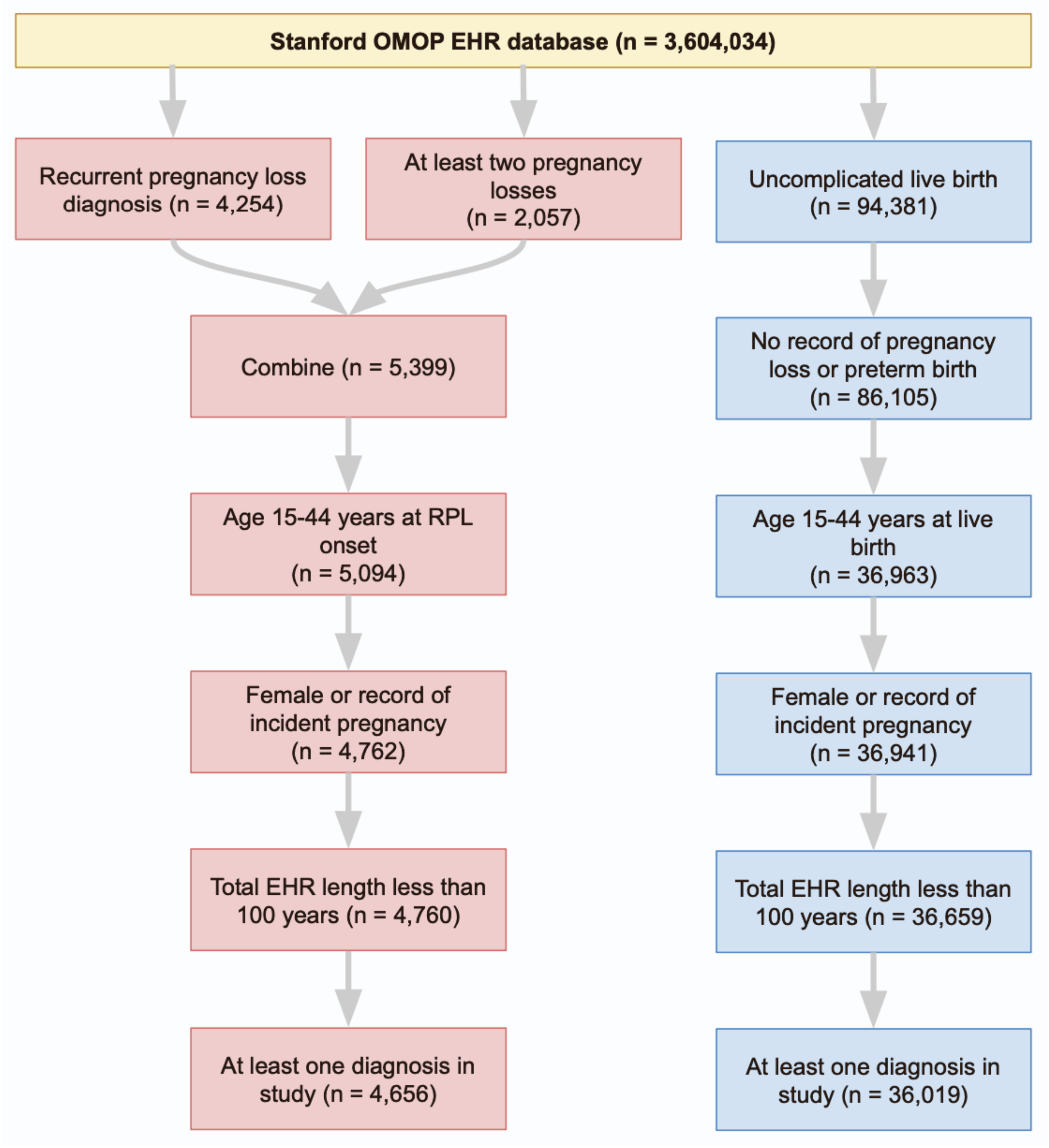

Supplementary figure 2: UCSF UMAPs by race and ethnicity.

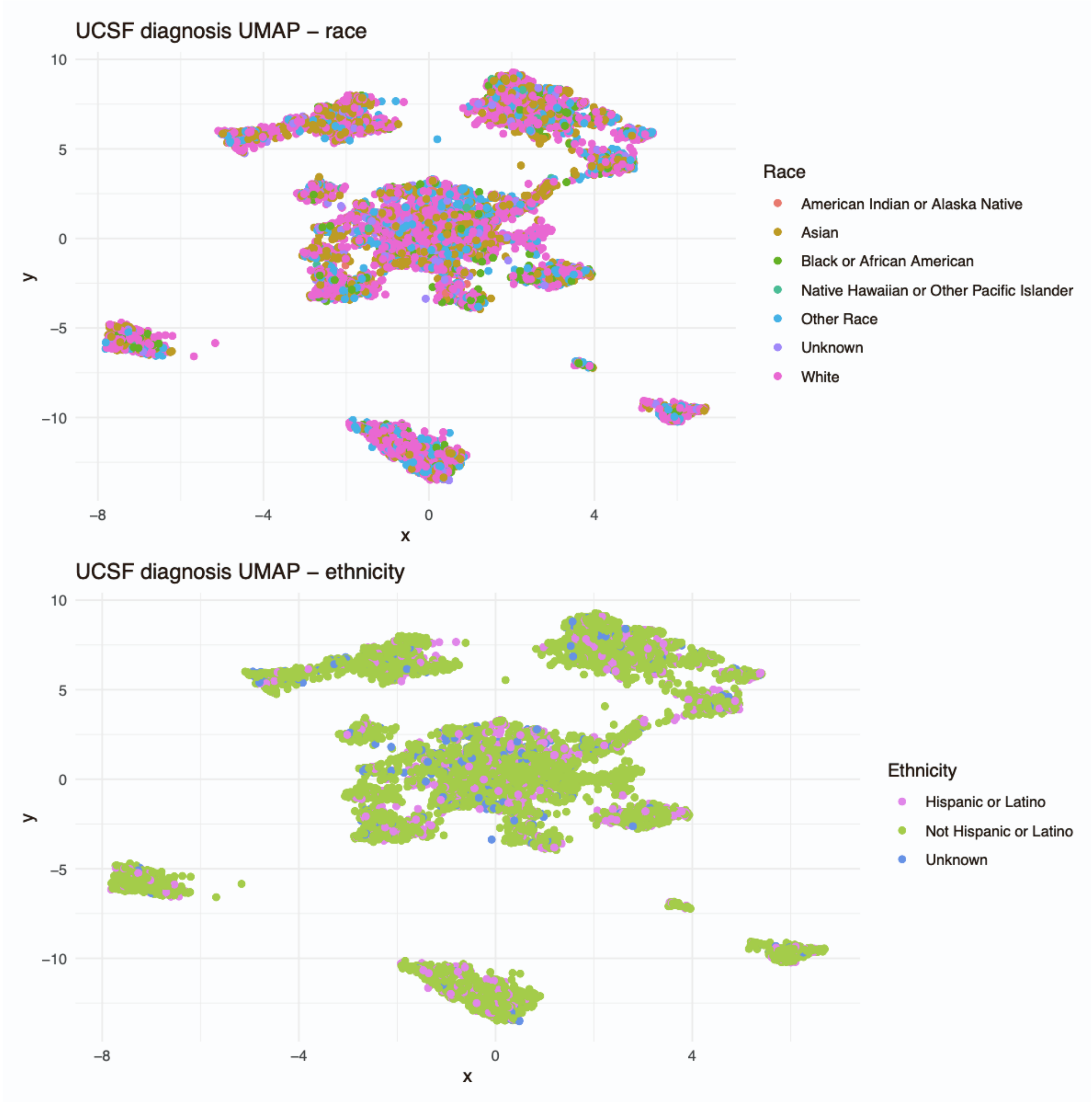

Supplementary figure 3: UCSF UMAPs by number of visits, years in EHR, and number of diagnoses.

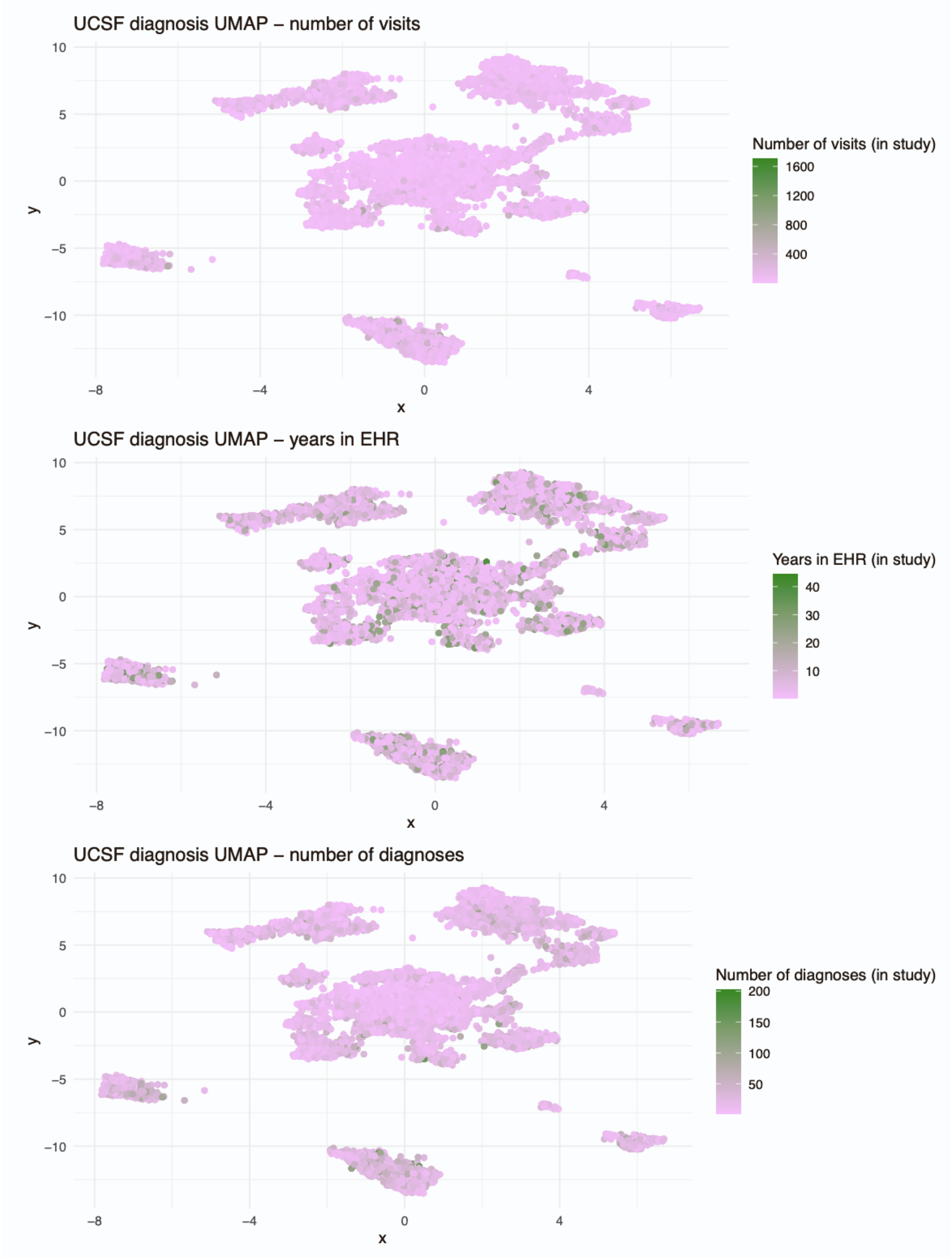

Supplementary figure 4: Stanford UMAPs by race and ethnicity.

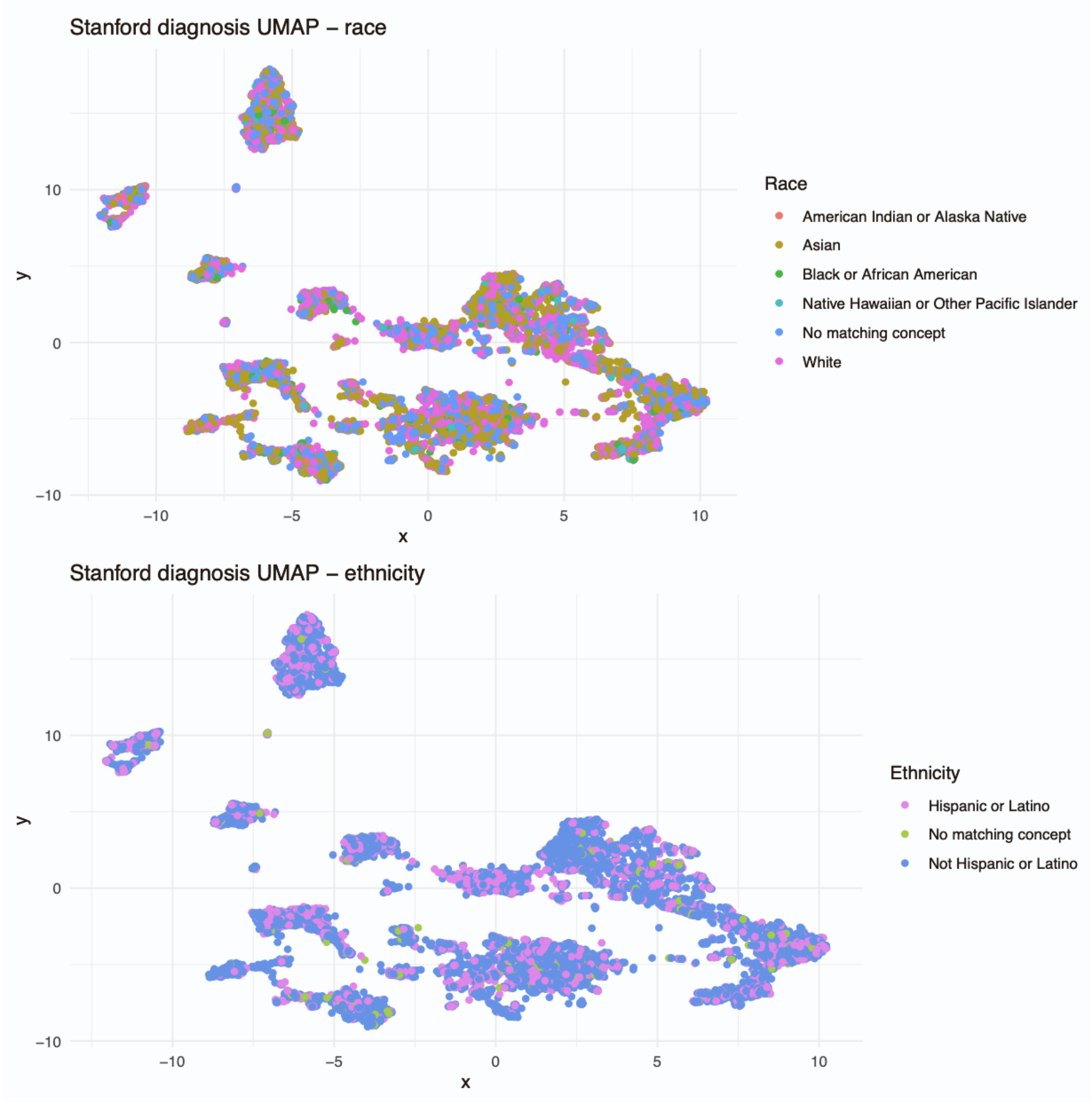

Supplementary figure 5: Stanford UMAPs by number of visits, years in EHR, and number of diagnoses.

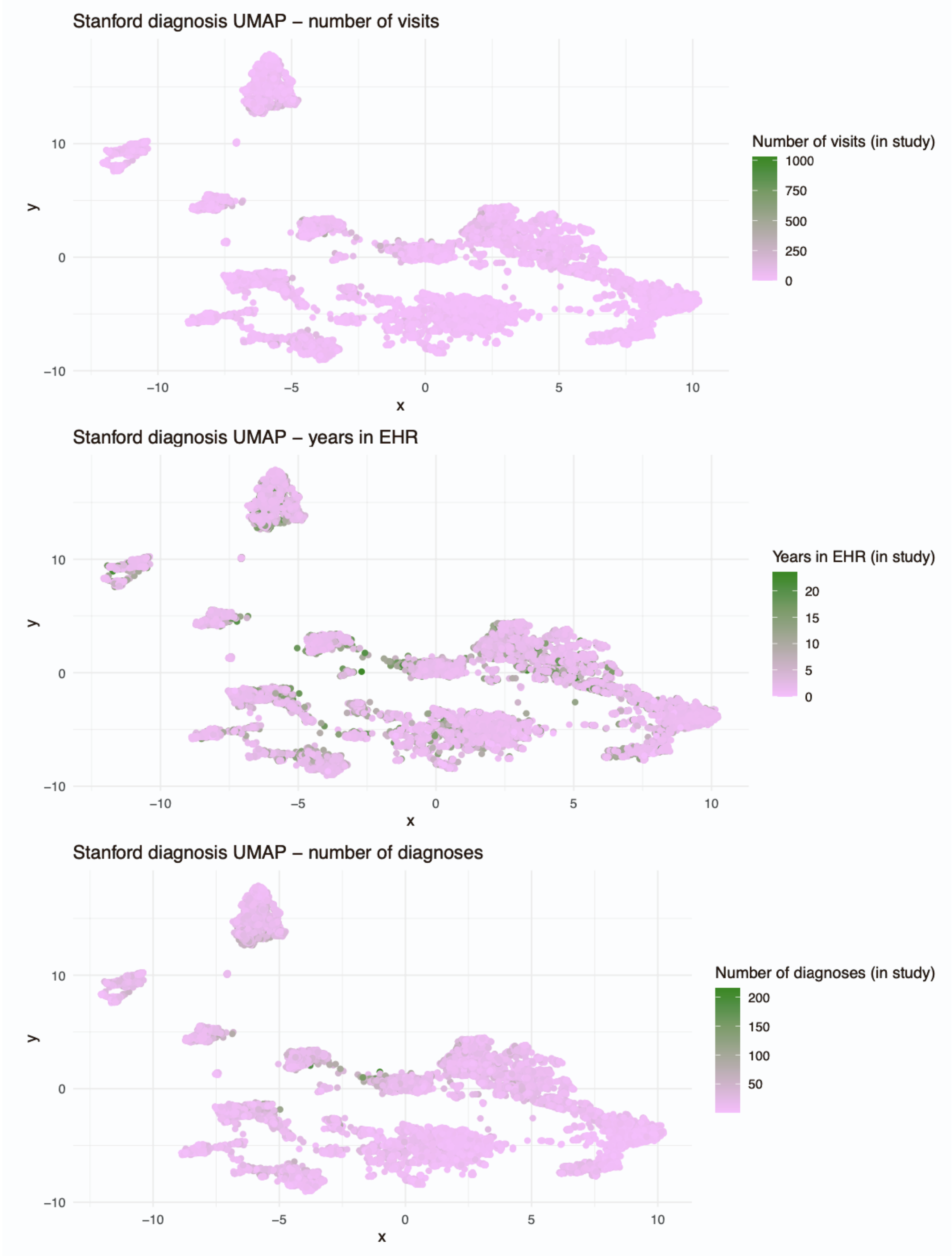

Supplementary figure 6: Association analysis results for Stanford patients: (a) all associations and (b) significant associations.

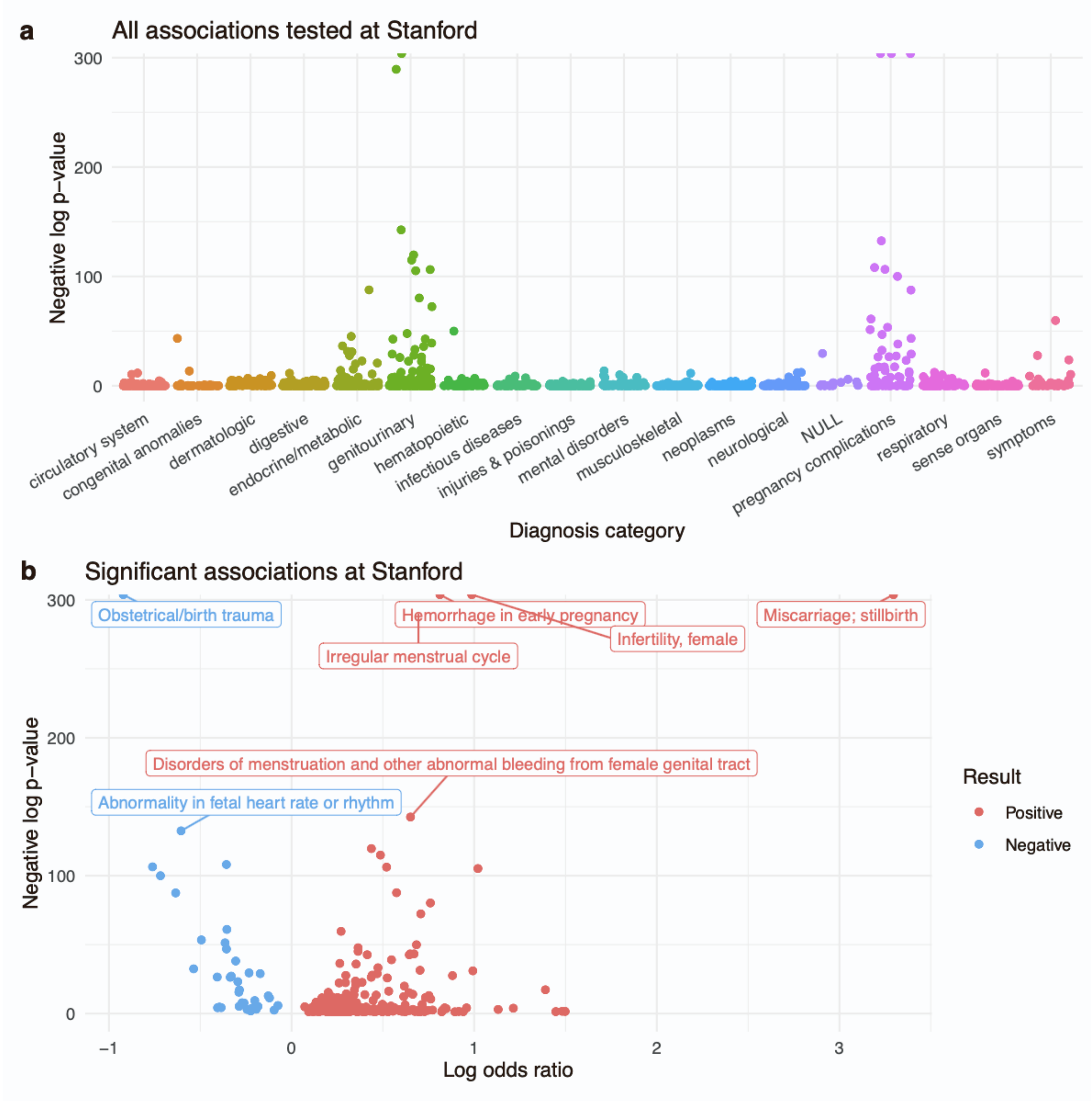

Supplementary figure 7: All significant results from Stanford age-stratified analysis.

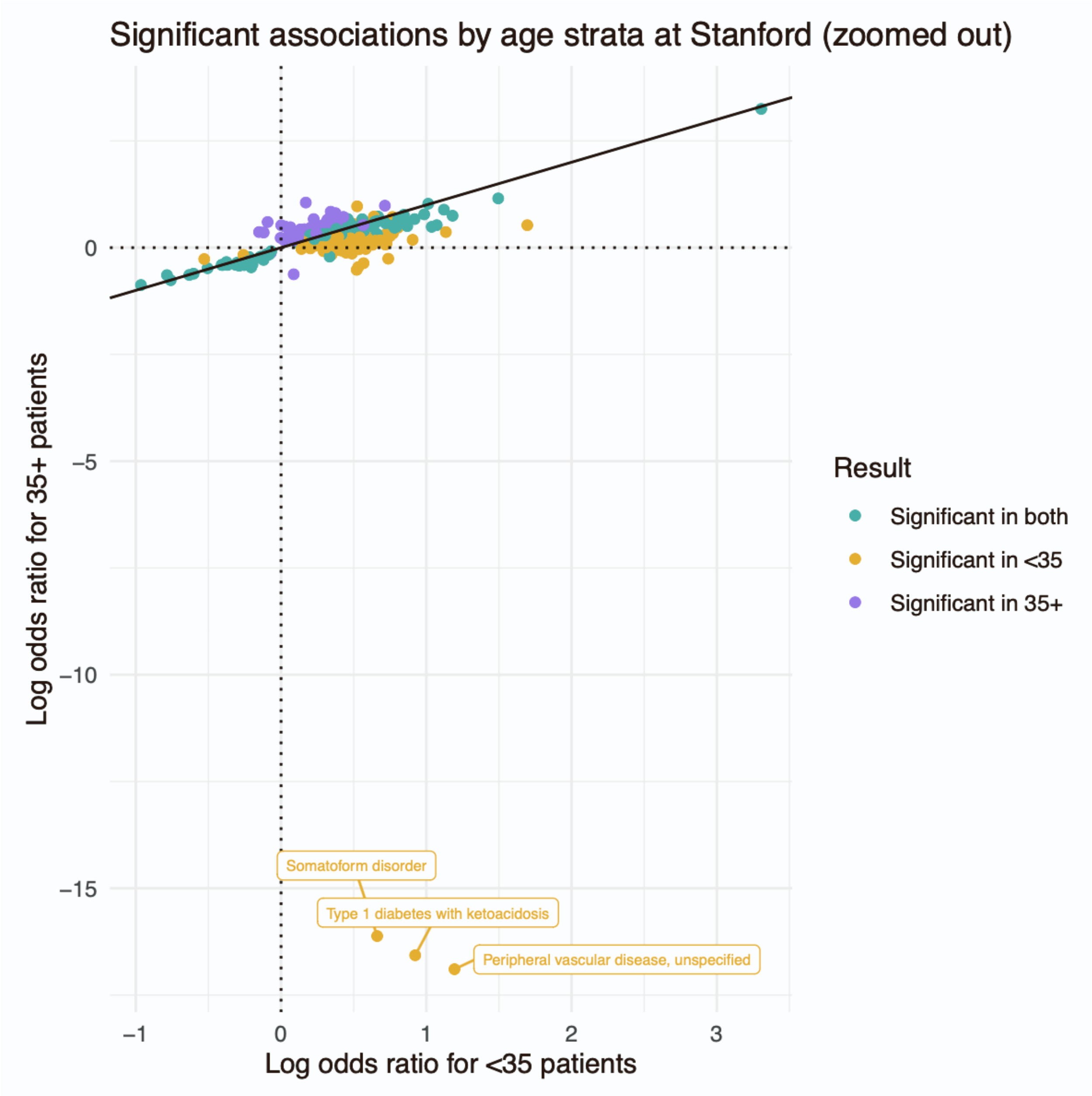

Supplementary table 1: Stanford demographics and healthcare utilization, all patients.

	RPL (N=4656)	Control (N=36019)	Total (N=40675)	P-value
**Age**				
Mean (SD)	35.1 (4.86)	31.9 (5.46)	32.2 (5.49)	<0.001
Median [Min, Max]	35.4 [17.1, 44.0]	32.4 [15.0, 44.0]	32.8 [15.0, 44.0]	
**Race**				
American Indian or Alaska Native	16 (0.3%)	118 (0.3%)	134 (0.3%)	<0.001
Asian	1600 (34.4%)	11198 (31.1%)	12798 (31.5%)	
Black or African American	143 (3.1%)	1039 (2.9%)	1182 (2.9%)	
Native Hawaiian or Other Pacific Islander	75 (1.6%)	713 (2.0%)	788 (1.9%)	
No matching concept	982 (21.1%)	8779 (24.4%)	9761 (24.0%)	
White	1840 (39.5%)	14172 (39.3%)	16012 (39.4%)	
**Ethnicity**				
Hispanic or Latino	763 (16.4%)	10552 (29.3%)	11315 (27.8%)	<0.001
No matching concept	243 (5.2%)	757 (2.1%)	1000 (2.5%)	
Not Hispanic or Latino	3650 (78.4%)	24710 (68.6%)	28360 (69.7%)	
**Number of visits (in study)**				
Mean (SD)	44.3 (46.3)	23.6 (29.6)	26.0 (32.6)	<0.001
Median [Min, Max]	31.0 [0, 544]	14.0 [0, 1030]	16.0 [0, 1030]	
**Years in EHR (in study)**				
Mean (SD)	4.97 (4.65)	4.55 (4.84)	4.59 (4.82)	<0.001
Median [Min, Max]	3.14 [0, 23.4]	1.67 [0, 23.6]	1.83 [0, 23.6]	
**Number of diagnoses (in study)**				
Mean (SD)	15.8 (15.7)	12.2 (11.1)	12.6 (11.8)	<0.001
Median [Min, Max]	11.0 [1.00, 202]	9.00 [1.00, 216]	9.00 [1.00, 216]	

Supplementary table 2: UCSF demographics and healthcare utilization, patients < 35.

	RPL (N=1401)	Control (N=11012)	Total (N=12413)	P-value
**Age**				
Mean (SD)	31.5 (3.24)	30.2 (4.06)	30.3 (4.00)	<0.001
Median [Min, Max]	32.4 [16.6, 35.0]	31.4 [15.1, 35.0]	31.5 [15.1, 35.0]	
**Race**				
American Indian or Alaska Native	17 (1.2%)	64 (0.6%)	81 (0.7%)	<0.001
Asian	259 (18.5%)	2386 (21.7%)	2645 (21.3%)	
Black or African American	97 (6.9%)	699 (6.3%)	796 (6.4%)	
Native Hawaiian or Other Pacific Islander	7 (0.5%)	82 (0.7%)	89 (0.7%)	
Other Race	243 (17.3%)	2414 (21.9%)	2657 (21.4%)	
Unknown	144 (10.3%)	679 (6.2%)	823 (6.6%)	
White	634 (45.3%)	4688 (42.6%)	5322 (42.9%)	
**Ethnicity**				
Hispanic or Latino	195 (13.9%)	2066 (18.8%)	2261 (18.2%)	<0.001
Not Hispanic or Latino	1073 (76.6%)	8200 (74.5%)	9273 (74.7%)	
Unknown	133 (9.5%)	746 (6.8%)	879 (7.1%)	
**Number of visits (in study)**				
Mean (SD)	58.2 (80.2)	47.4 (48.0)	48.6 (52.8)	<0.001
Median [Min, Max]	36.0 [1.00, 1700]	37.0 [1.00, 1230]	37.0 [1.00, 1700]	
**Years in EHR (in study)**				
Mean (SD)	5.16 (6.62)	4.59 (5.98)	4.65 (6.05)	0.00229
Median [Min, Max]	2.37 [0.225, 44.4]	1.69 [0.132, 38.9]	1.77 [0.132, 44.4]	
**Number of diagnoses (in study)**				
Mean (SD)	13.6 (16.0)	14.1 (11.3)	14.0 (11.9)	0.303
Median [Min, Max]	8.00 [1.00, 202]	12.0 [1.00, 186]	12.0 [1.00, 202]	

Supplementary table 3: UCSF demographics and healthcare utilization, patients 35+.

	RPL (N=2439)	Control (N=6247)	Total (N=8686)	P-value
**Age**				
Mean (SD)	38.9 (2.41)	37.9 (2.15)	38.2 (2.28)	<0.001
Median [Min, Max]	38.9 [35.0, 44.0]	37.4 [35.0, 44.0]	37.7 [35.0, 44.0]	
**Race**				
American Indian or Alaska Native	16 (0.7%)	22 (0.4%)	38 (0.4%)	<0.001
Asian	566 (23.2%)	1486 (23.8%)	2052 (23.6%)	
Black or African American	83 (3.4%)	179 (2.9%)	262 (3.0%)	
Native Hawaiian or Other Pacific Islander	19 (0.8%)	63 (1.0%)	82 (0.9%)	
Other Race	327 (13.4%)	987 (15.8%)	1314 (15.1%)	
Unknown	222 (9.1%)	364 (5.8%)	586 (6.7%)	
White	1206 (49.4%)	3146 (50.4%)	4352 (50.1%)	
**Ethnicity**				
Hispanic or Latino	255 (10.5%)	803 (12.9%)	1058 (12.2%)	<0.001
Not Hispanic or Latino	1949 (79.9%)	5066 (81.1%)	7015 (80.8%)	
Unknown	235 (9.6%)	378 (6.1%)	613 (7.1%)	
**Number of visits (in study)**				
Mean (SD)	72.6 (79.0)	63.2 (57.6)	65.9 (64.5)	<0.001
Median [Min, Max]	48.0 [1.00, 1110]	50.0 [1.00, 635]	49.0 [1.00, 1110]	
**Years in EHR (in study)**				
Mean (SD)	6.47 (6.32)	5.37 (5.70)	5.68 (5.90)	<0.001
Median [Min, Max]	4.15 [0.137, 37.7]	2.74 [0.712, 41.4]	3.13 [0.137, 41.4]	
**Number of diagnoses (in study)**				
Mean (SD)	13.7 (14.8)	16.9 (12.0)	16.0 (12.9)	<0.001
Median [Min, Max]	9.00 [1.00, 162]	14.0 [1.00, 126]	13.0 [1.00, 162]	

Supplementary table 4: Stanford demographics and healthcare utilization, patients < 35.

	RPL (N=2194)	Control (N=25343)	Total (N=27537)	P-value
**Age**				
Mean (SD)	31.0 (3.38)	29.4 (4.35)	29.5 (4.30)	<0.001
Median [Min, Max]	31.8 [17.1, 35.0]	30.5 [15.0, 35.0]	30.6 [15.0, 35.0]	
**Race**				
American Indian or Alaska Native	7 (0.3%)	91 (0.4%)	98 (0.4%)	<0.001
Asian	742 (33.8%)	7260 (28.6%)	8002 (29.1%)	
Black or African American	83 (3.8%)	826 (3.3%)	909 (3.3%)	
Native Hawaiian or Other Pacific Islander	37 (1.7%)	535 (2.1%)	572 (2.1%)	
No matching concept	503 (22.9%)	6894 (27.2%)	7397 (26.9%)	
White	822 (37.5%)	9737 (38.4%)	10559 (38.3%)	
**Ethnicity**				
Hispanic or Latino	439 (20.0%)	8364 (33.0%)	8803 (32.0%)	<0.001
No matching concept	106 (4.8%)	524 (2.1%)	630 (2.3%)	
Not Hispanic or Latino	1649 (75.2%)	16455 (64.9%)	18104 (65.7%)	
**Number of visits (in study)**				
Mean (SD)	39.1 (40.9)	21.9 (25.3)	23.2 (27.3)	<0.001
Median [Min, Max]	28.0 [0, 474]	14.0 [0, 466]	14.0 [0, 474]	
**Years in EHR (in study)**				
Mean (SD)	4.51 (4.55)	4.33 (4.75)	4.35 (4.73)	0.0746
Median [Min, Max]	2.51 [0, 23.4]	1.63 [0, 23.6]	1.64 [0, 23.6]	
**Number of diagnoses (in study)**				
Mean (SD)	15.6 (15.7)	11.8 (10.7)	12.1 (11.2)	<0.001
Median [Min, Max]	11.0 [1.00, 202]	9.00 [1.00, 188]	9.00 [1.00, 202]	

Supplementary table 5: Stanford demographics and healthcare utilization, patients 35+.

	RPL (N=2462)	Control (N=10676)	Total (N=13138)	P-value
**Age**				
Mean (SD)	38.7 (2.44)	37.9 (2.15)	38.0 (2.24)	<0.001
Median [Min, Max]	38.5 [35.0, 44.0]	37.4 [35.0, 44.0]	37.6 [35.0, 44.0]	
**Race**				
American Indian or Alaska Native	9 (0.4%)	27 (0.3%)	36 (0.3%)	0.114
Asian	858 (34.8%)	3938 (36.9%)	4796 (36.5%)	
Black or African American	60 (2.4%)	213 (2.0%)	273 (2.1%)	
Native Hawaiian or Other Pacific Islander	38 (1.5%)	178 (1.7%)	216 (1.6%)	
No matching concept	479 (19.5%)	1885 (17.7%)	2364 (18.0%)	
White	1018 (41.3%)	4435 (41.5%)	5453 (41.5%)	
**Ethnicity**				
Hispanic or Latino	324 (13.2%)	2188 (20.5%)	2512 (19.1%)	<0.001
No matching concept	137 (5.6%)	233 (2.2%)	370 (2.8%)	
Not Hispanic or Latino	2001 (81.3%)	8255 (77.3%)	10256 (78.1%)	
**Number of visits (in study)**				
Mean (SD)	48.9 (50.1)	27.9 (37.5)	31.8 (41.0)	<0.001
Median [Min, Max]	34.0 [0, 544]	16.0 [0, 1030]	18.0 [0, 1030]	
**Years in EHR (in study)**				
Mean (SD)	5.37 (4.70)	5.06 (5.04)	5.12 (4.98)	0.00311
Median [Min, Max]	3.85 [0, 23.4]	2.32 [0, 23.5]	2.60 [0, 23.5]	
**Number of diagnoses (in study)**				
Mean (SD)	16.1 (15.7)	13.2 (12.0)	13.7 (12.8)	<0.001
Median [Min, Max]	11.0 [1.00, 169]	10.0 [1.00, 216]	10.0 [1.00, 216]	

Methods S1

Time window sensitivity analysis

To test whether our main findings, menstrual abnormalities and infertility diagnoses, are sensitive to the inclusion of the 1-year window following RPL or live birth, we repeated our main analysis but restricted it to diagnoses occurring before RPL or live birth (respective t0s). In this sensitivity analysis, 55 diagnoses are significantly positively associated with RPL, of which 29 are also significantly positively associated with RPL in the main analysis. Several menstrual abnormalities and infertility diagnoses remain significantly positively associated with RPL in this sensitivity analysis, including: excessive or frequent menstruation, dysmenorrhea, irregular menstrual cycle/bleeding, disorders of menstruation and other abnormal bleeding from female genital tract, female infertility, anovulation, ovarian dysfunction, ovarian failure, PCOS, and endometriosis. Overall, these results suggest that our main findings, menstrual abnormalities and infertility diagnoses, are not sensitive to inclusion of the 1-year window. Association results from this analysis are reported in supplementary file 13.

Prior live birth sensitivity analysis

To test whether the findings from our next pregnancy analysis are sensitive to prior live births, we re-estimated associations with adjustment for prior live birth (yes/no) at the medical center. Among the RPL patients who went on to have a live birth in their next pregnancy, 142 have a prior live birth and 617 do not. Among the RPL patients who went on to have a pregnancy loss in their next pregnancy, 95 have a prior live birth and 639 do not. The results from this sensitivity analysis are extremely similar to the results from the next pregnancy analysis. The same 1 diagnosis (metabolic syndrome) is significantly positively associated with loss. Of the 48 diagnoses that are significantly negatively associated with loss in the next pregnancy analysis, 47 are also significantly negatively associated with loss in this sensitivity analysis. Only 1 diagnosis (miscarriage or stillbirth) has a different result in this sensitivity analysis, however the directionality is the same and the *p*-value (0.08) is just above our threshold for statistical significance. Overall, these results suggest that the findings in the next pregnancy analysis are not sensitive to adjustment for prior live birth. Association results from this analysis are reported in supplementary file 14.

Propensity score matching sensitivity analysis

To test whether our association results are sensitive to the method used for addressing potential confounding, we performed propensity score matching on age, race, and ethnicity using the R package MatchIt. A subset of 3840 control patients were selected as a 1:1 matched control group for the 3840 RPL patients. The resulting RPL and control groups are well-balanced; all of the absolute standardized mean differences between the matched groups' age, race, and ethnicity are less than 0.05. We then carried out our association analysis using the same

methods as in the main analysis and compared results. In this propensity score matching sensitivity analysis, 40 diagnoses are significantly positively associated with RPL, of which 39 are also significantly positively associated with RPL in the main analysis. The one diagnosis that is not also significantly positively associated with RPL in the main analysis is “Noninflammatory disorders of ovary, fallopian tube, and broad ligament”. However, the directionality of effect is the same (odds ratio: 1.33, *p*-value: 0.12 in main analysis). Association results from this analysis are reported in supplementary file 15.

Infertility sensitivity analysis

Given that RPL patients may be more likely to experience infertility and infertility patients may have increased diagnostic opportunities during assisted reproductive care such as in vitro fertilization, we carried out a sensitivity analysis where patients with an infertility diagnosis during the study window (anytime before the index date up until a year after the index date) were excluded. The excluded OMOP concept ids were: 201909, 197044, 200152, 197606, 201635, 194419, and 198197. In total, 1,562 RPL patients and 1,521 control patients had an infertility concept recorded and were subsequently removed for this analysis. 66 diagnoses were significantly positively associated with RPL; 43 were also RPL-associated in the main analysis. All results are in supplementary file 16. Similar to the main analysis, association results from this sensitivity analysis included menstrual abnormalities, polycystic ovaries, endometriosis, and metabolic syndrome.

Sensitivity analysis of <35 patients adjusted for healthcare utilization

To explore further our findings that effect sizes were larger among patients aged 35 years or younger, we additionally estimated diagnostic associations for that age group with healthcare utilization (measured via number of visits in study window) included as a covariate in the model. 38 diagnoses were significantly positively associated with RPL, and 36 of them were also significantly positively associated with RPL in the main analysis. Odds ratios were higher in the sensitivity analysis than in the main analysis for 32 of 36 diagnoses.

Supplementary Figure Captions

Supplementary Figure 1: Patient selection at Stanford. In total, 4,656 RPL patients were selected (red) and 36,019 control patients were selected (blue).

Supplementary Figure 2: UCSF diagnosis UMAPs colored by: **(a)** race and **(b)** ethnicity.

Supplementary Figure 3: UCSF diagnosis UMAPs colored by: **(a)** years in EHR, **(b)** number of visits, and **(c)** number of diagnoses.

Supplementary Figure 4: Stanford diagnosis UMAPs colored by: **(a)** race and **(b)** ethnicity.

Supplementary Figure 5: Stanford diagnosis UMAPs colored by: **(a)** years in EHR, **(b)** number of visits, and **(c)** number of diagnoses.

Supplementary Figure 6: Stanford association analysis results. **(a)** Manhattan plot of all diagnoses tested in the UCSF analysis. Diagnosis categories are listed on the x-axis. The y-axis is the negative log of each associations' *p*-value, from each diagnosis's GAM. The NULL-labeled category contains diagnoses that were not part of any of the other categories listed. **(b)** Volcano plot of significant (*p*-value<0.05) associations. In the manhattan and volcano plots, a few diagnoses have negative log *p*-values that are approaching infinity, so their corresponding points are located on the top border of the plot. All log transformations in these plots are in base 10.

Supplementary Figure 7: Full log-log plot from age-stratified analysis at Stanford. The zoomed-in version of this plot is in Figure 5b. All points in the log-log plot were filtered to include diagnoses where ≥10 patients in either the RPL or control group have a record of that diagnosis. All log transformations in these plots are in base 10.

Supplementary Table Captions

Supplementary Table 1: Patient demographics and healthcare utilization at Stanford. *P*-values were computed using chi-squared tests for categorical variables and two-sided t-tests for numeric variables.

Supplementary Table 2: Patient demographics and healthcare utilization for the <35 stratum in the UCSF age-stratified analysis. *P*-values were computed using chi-squared tests for categorical variables and two-sided t-tests for continuous variables.

Supplementary Table 3: Patient demographics and healthcare utilization for the 35+ stratum in the UCSF age-stratified analysis. *P*-values were computed using chi-squared tests for categorical variables and two-sided t-tests for continuous variables.

Supplementary Table 4: Patient demographics and healthcare utilization for the <35 stratum in the Stanford age-stratified analysis. *P*-values were computed using chi-squared tests for categorical variables and two-sided t-tests for continuous variables.

Supplementary Methods

Methods S1: Supplementary Methods
